# Heterodimer formation with retinoic acid receptor RXRα modulates coactivator recruitment by peroxisome proliferator-activated receptor PPARγ

**DOI:** 10.1016/j.jbc.2021.100814

**Published:** 2021-05-31

**Authors:** Whitney Kilu, Daniel Merk, Dieter Steinhilber, Ewgenij Proschak, Jan Heering

**Affiliations:** 1 Institute of Pharmaceutical Chemistry, Goethe University Frankfurt, Frankfurt, Germany; 2 Assay development and screening, Fraunhofer Institute for Translational Medicine and Pharmacology ITMP, Frankfurt, Germany

**Keywords:** PPARγ, RXRα, heterodimer, homodimer, homogeneous time-resolved FRET (HTRF), cofactor recruitment, allostery, drug target, AF-2, activation function 2, CBP-1, CREB-binding protein coactivator motif 1, DBD, DNA-binding domain, FXR, farnesoid X receptor, HTRF, homogeneous time-resolved FRET, LBD, ligand-binding domain, NR, nuclear receptor, PPAR, peroxisome proliferator-activated receptor, RXR, retinoid X receptor, sGFP, super folder green fluorescent protein, SRC1-2, steroid receptor coactivator 1 motif 2, Tb-SA, terbium cryptate conjugated to streptavidin, TETRAC, non-classical thyroid hormone 3,3',5,5'-tetraiodothyroacetic acid

## Abstract

Nuclear receptors (NRs) activate transcription of target genes in response to binding of ligands to their ligand-binding domains (LBDs). Typically, *in vitro* assays use either gene expression or the recruitment of coactivators to the isolated LBD of the NR of interest to measure NR activation. However, this approach ignores that NRs function as homo- as well as heterodimers and that the LBD harbors the main dimerization interface. Cofactor recruitment is thereby interconnected with oligomerization status as well as ligand occupation of the partnering LBD through allosteric cross talk. Here we present a modular set of homogeneous time-resolved FRET–based assays through which we investigated the activation of PPARγ in response to ligands and the formation of heterodimers with its obligatory partner RXRα. We introduced mutations into the RXRα LBD that prevent coactivator binding but do not interfere with LBD dimerization or ligand binding. This enabled us to specifically detect PPARγ coactivator recruitment to PPARγ:RXRα heterodimers. We found that the RXRα agonist SR11237 destabilized the RXRα homodimer but promoted formation of the PPARγ:RXRα heterodimer, while being inactive on PPARγ itself. Of interest, incorporation of PPARγ into the heterodimer resulted in a substantial gain in affinity for coactivator CBP-1, even in the absence of ligands. Consequently, SR11237 indirectly promoted coactivator binding to PPARγ by shifting the oligomerization preference of RXRα toward PPARγ:RXRα heterodimer formation. These results emphasize that investigation of ligand-dependent NR activation should take NR dimerization into account. We envision these assays as the necessary assay tool kit for investigating NRs that partner with RXRα.

Peroxisome proliferator-activated receptors (PPARs) are important targets for approved and experimental drugs in many clinical indications, including metabolic (1, 2) and inflammatory diseases (3, 4). The three PPAR subtypes, PPARα, PPARγ, and PPARβ/δ form heterodimers with their obligatory dimer partner retinoid X receptor (RXR) (5). Peroxisome proliferator-activated receptor γ (PPARγ) is the most explored of the three subtypes being in the focus of biochemical, structural, and pharmaceutical research for decades. After almost all approved PPARγ ligands from the thiazolidinone class were withdrawn from the market owing to adverse effects, a variety of modulators that overcome the drawbacks of classical full agonists were developed. In terms of pharmacology, partial agonists and antagonists have been developed, paving the way for a resurrection of PPARγ as an attractive pharmacological target.

However, current pharmacological models do not fully reflect the complexity of PPARγ modulation by various ligands. Heterodimers formed between PPARγ and its obligatory dimer partner retinoid X receptor α (RXRα) can activate transcription of target genes in response to ligands specific to either of the dimer partners’ ligand-binding domain (LBD).

Hence, modulation of heterodimer formation has major influence on target gene expression or repression and thus modulates the gene expression in response to ligands. Dissociation of the RXR:RXR homodimer allows for the formation of the PPARγ:RXR heterodimer. But as RXR is also a potential heterodimer partner for a variety of other nuclear receptors (21 in case of RXRα (5)) these nuclear receptors compete for dimerization with RXR and, hence, influence its availability. Just recently, such crisscross competition for RXRα was shown for PPARγ, RARα, and VDR (6). Once the PPARγ:RXR heterodimer is formed, different corepressors and coactivators are recruited to the heterodimer. Each step of this complex interplay between RXR and PPARγ can be modulated by a ligand acting either on one or the other heterodimer partner or on both partners simultaneously.

Several examples illustrate the complexity of the underlying modulation of the PPARγ:RXR heterodimer. It has been reported on the biological characterization of a heterodimer-selective RXR modulator which holds potential for RXR targeting in metabolic indications (7). Additional beneficial effects on Kupffer cells in the context of hepatic injury were observed upon simultaneous activation of PPARγ and RXR by the corresponding agonists (8). Furthermore, insulin sensitizing effects were documented for the RXR ligand LG100754, which antagonizes the transactivation of target genes of the RXR:RXR homodimer but acts as an agonist on heterodimers of RXR with PPARα, PPARγ, or PPARδ (9) as well as RAR (10). These studies demonstrate that simultaneous treatment with agonists specific for both PPARγ and RXR may result in a synergistic pharmacological effect.

Classical assay technologies for nuclear receptors that are used for screening and ligand characterization in drug discovery often rely either on cofactor recruitment to the recombinantly expressed isolated LBD or on transactivation of reporter genes by Gal4-LBD chimera. In this type of assays the activity of the nuclear receptor (NR) in terms of coactivator recruitment or transactivation of reporter genes is detected independently of the oligomerization status of the LBD. Thus, it is not possible to differentiate if detected activity relates to monomeric LBD or to LBD dimers. In addition, these assays lack the possibility to study how ligands modulate the complex process of RXR:RXR homodimer dissociation and heterodimer formation.

In this study we provide insights into how diverse agonists and antagonists of RXR and PPAR modulate formation and activation of the RXR:PPAR heterodimer, which holds important implications for future drug discovery targeting nuclear receptors.

## Results

### RXRα LBD forms heterodimers preferentially with PPARγ LBD

**Figure 1:**
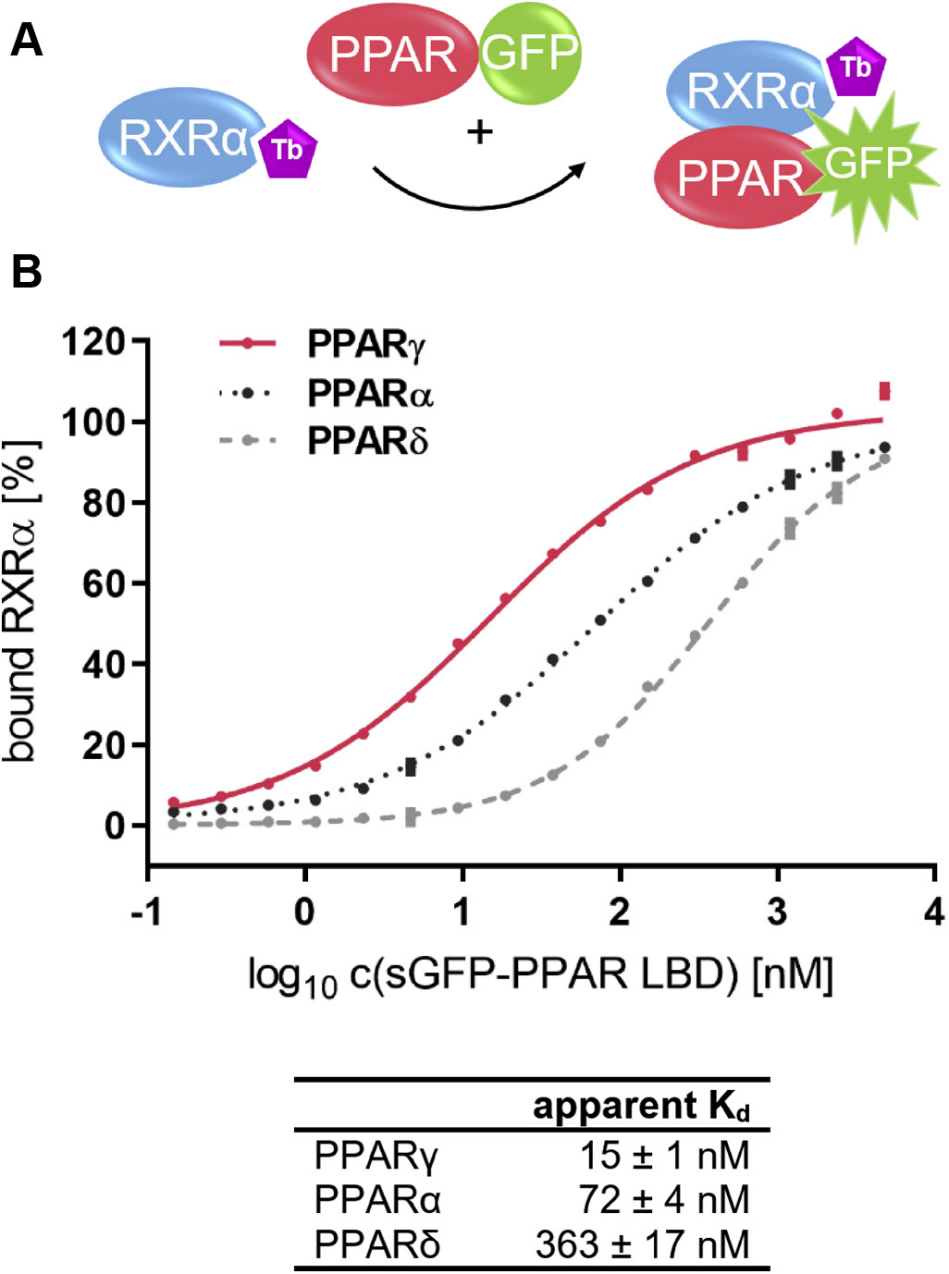
**Heterodimer formation between RXRα LBD and the LBDs of PPARα, γ and δ.***A*, schematic representation of the heterodimer formation assay with RXRα LBD being coupled to the FRET donor. *B*, the LBD of RXRα was labeled with biotin *via* an N-terminal Avi-tag and coupled to Tb-SA (0.375 nM RXRα and 0.75 nM Streptavidin monomers, respectively). The PPAR LBDs as fusion proteins with N-terminal sGFP were titrated up to 4.8 μM. By addition of free sGFP the total concentration of sGFP was kept constant throughout the entire experiment. Data are the mean ± SD; N = 4. R^2^ for each curve equals >99%. Curves were fitted based on the HTRF signals before conversion to bound RXR [%] for better comparison. The lower and upper plateaus from the fits were set as 0% and 100% bound, respectively. LBD, ligand-binding domain; PPAR, peroxisome proliferator-activated receptor; RXR, retinoid X receptor.

To probe the relative affinities of the three PPAR subtypes to RXRα, we constructed a homogeneous time-resolved FRET (HTRF)-based assay for investigation of heterodimer formation between the LBDs of the PPARs and the LBD of their obligatory dimer partner RXRα. RXRα was labeled with biotin *via* an N-terminal Avi-tag and subsequently coupled to streptavidin labeled with Terbium cryptate (Tb-SA). The latter was utilized as the FRET donor fluorophore. The PPAR LBDs were expressed with N-terminal superfolder green fluorescent protein (sGFP) that functions as the FRET acceptor. We then titrated sGFP-PPAR LBD to up to 4.8 μM until saturation in HTRF indicated maximal complex formation (Fig. 1).

In order to suppress secondary effects caused by variation of sGFP concentration and its influence on diffusion-enhanced FRET we used free sGFP to keep the total sGFP concentration constant at 4.8 μM. Proper dimer formation capability of all PPAR protein preparations was validated *via* a pulldown experiment (Figs. S1 and S2).

Separate control experiments showed that, in this experimental setup, an increase in the HTRF signal correlates with the binding of sGFP-PPAR to RXRα-LBD. When the latter was not coupled to Tb-SA owing to an excess of free biotin in the sample, then the HTRF signal obtained was the same as for the control with no RXRα being present (Fig. S3).

The three PPAR subtypes differed substantially in their observed affinity for binding to the RXRα LBD (*p* < 0.0001 in sum-of-squares F-test). PPARδ showed the lowest affinity with a *K*_*d*_ of 363 nM. Second in the order was PPARα with about five times higher affinity to RXRα (*K*_*d*_ = 72 nM). The highest affinity was observed for PPARγ with a *K*_*d*_ of 15 nM, which is another factor five higher. Since the heterodimer formation between RXRα and PPARγ exhibited the lowest dissociation constant we further concentrated on investigating factors that modulate this heterodimer.

### PPARγ agonists stabilize the heterodimer with RXRα

Next we investigated the influence of PPARγ ligands on the formation of the heterodimer with RXRα. To examine the effect of saturating the PPARγ LBD with any of its ligands, the PPARγ LBD was kept at a constant low concentration. Consequently, we inverted the setup and coupled PPARγ to the FRET donor. RXRα as a fusion protein with sGFP was titrated, whereas all other factors were kept constant. Again, separate control experiments were conducted, which provided evidence that an increase in HTRF correlated with the formation of the heterodimer (Fig. S4). And, when activated by its reference agonist rosiglitazone, the utilized biotin-labeled PPARγ recruited a fluorescein-labeled peptide derived from coactivator CBP-1 in a dose-dependent manner (Fig. S5). This showed that coupling to Tb-SA does not compromise the function of the LBD.

Incubation with a fixed concentration of 1 μM PPARγ full agonist GW1929, partial agonist INT131, or reference agonist rosiglitazone resulted in distinct shifts of the titration curves compared with the apo experiment (*p* < 0.0014 (INT131); *p* < 0.0001 (Rosi; GW1929) in sum-of-squares F-test).

**Figure 2:**
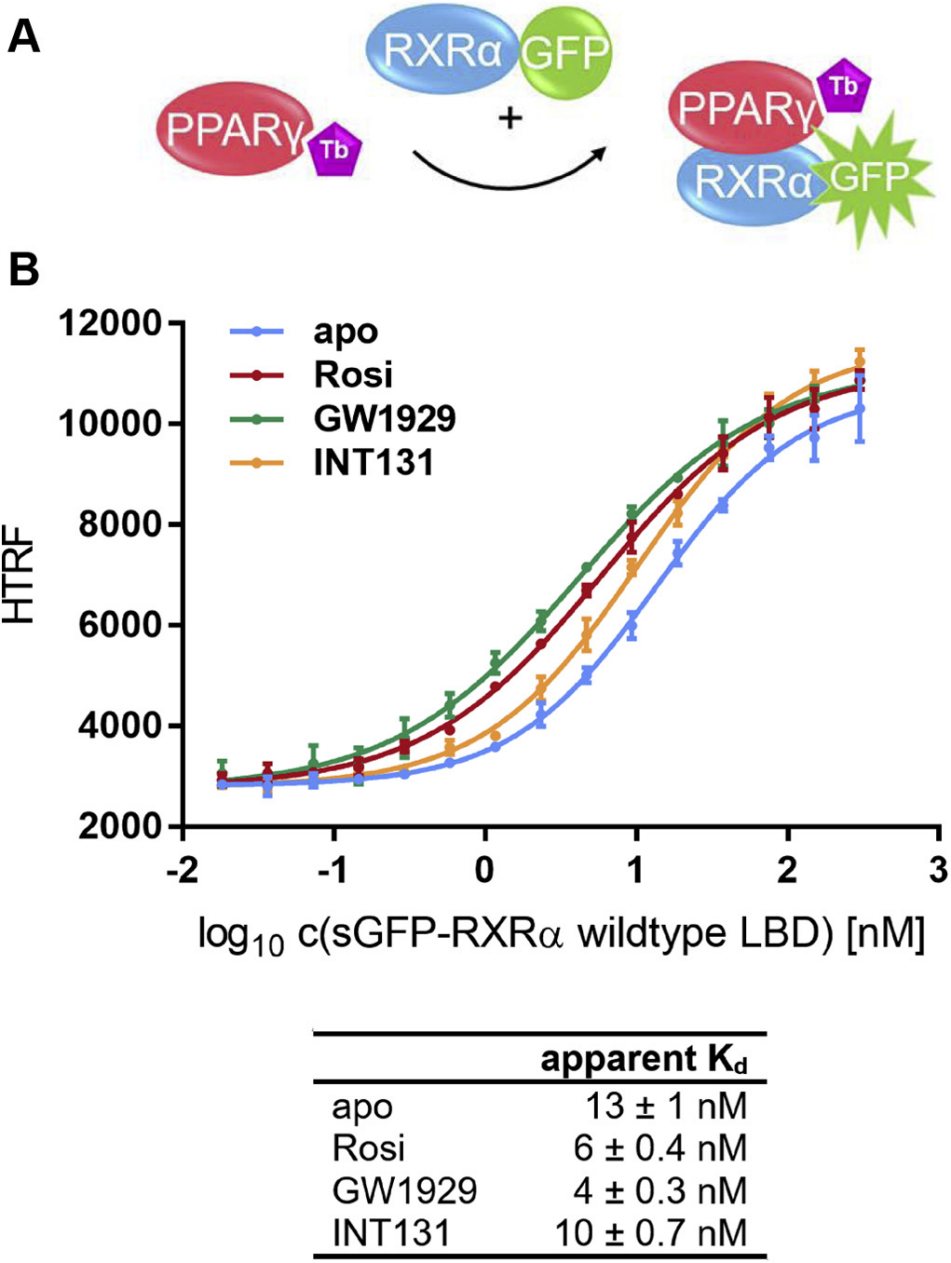
**Effect of PPARγ agonists on PPARγ-RXRα heterodimer formation.***A*, schematic representation of the heterodimer formation assay with PPARγ LBD being coupled to the FRET donor. *B*, sGFP-RXRα LBD was titrated against 0.375 nM biotinylated PPARγ LBD and 0.75 nM Tb-SA with either 1 μM PPARγ full agonist GW1929 (*green*), partial agonist INT131 (*yellow*), reference agonist rosiglitazone (*red*), or no ligand at all (*light blue*). By addition of free sGFP the total concentration of sGFP was kept constant at 0.3 μM throughout the entire experiment. Data are the mean ± SD; N = 3. R^2^ for each curve equals >99%. LBD, ligand-binding domain; PPAR, peroxisome proliferator-activated receptor; RXR, retinoid X receptor.

GW1929 caused a 3-fold reduction of the apparent *K*_*d*_ of heterodimer formation, thereby promoting the dimerization of the PPARγ LBD and the RXRα LBD. The reference agonist rosiglitazone reduced the *K*_*d*_ by a factor of 2. Incubation with INT131, which is a partial agonist of PPARγ, resulted in an only minor stabilization of the heterodimer (Fig. 2). The PPARγ antagonists SR1664 and GW9662 did not affect heterodimer formation (Fig. S6).

### RXRα agonist SR11237 reduces RXRα homodimer stability but promotes formation of the PPARγ:RXRα heterodimer

**Figure 3:**
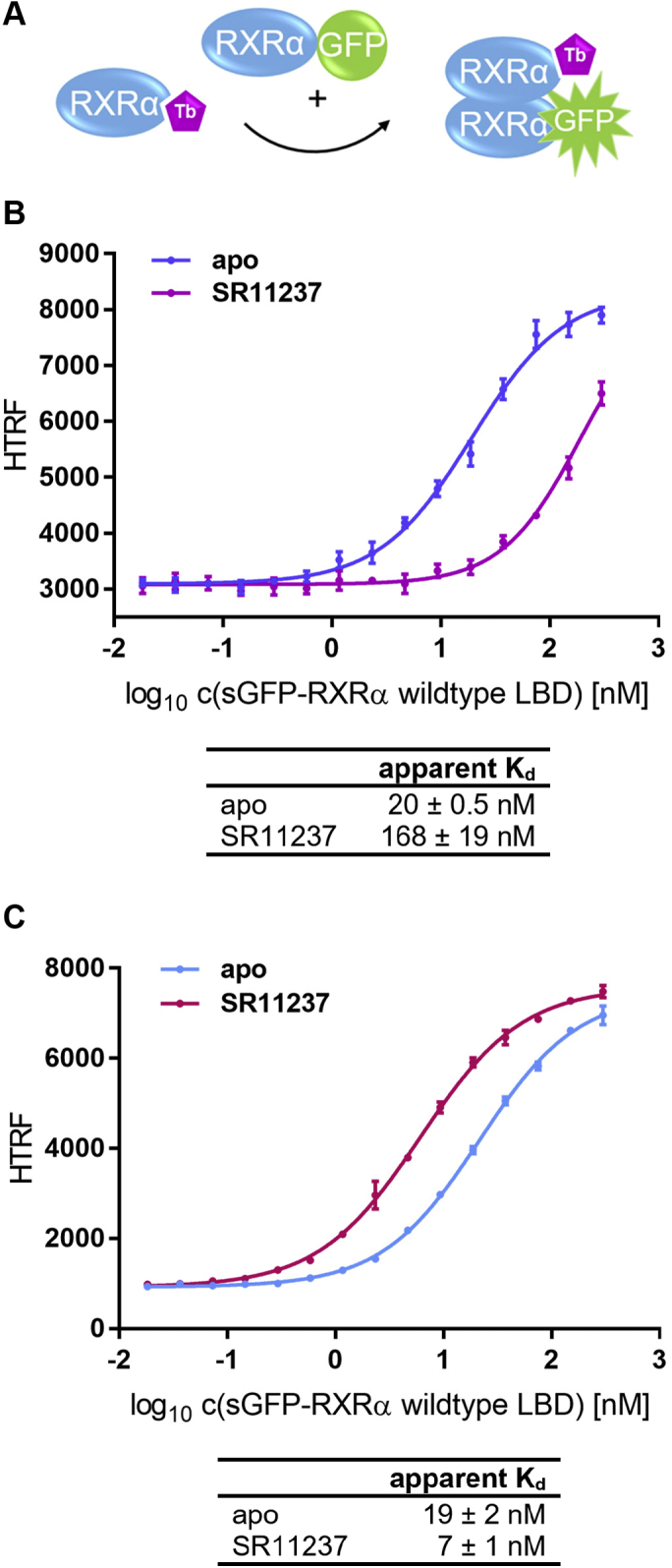
**Effect of RXRα agonist SR11237 on RXRα homodimer and PPARγ:RXRα heterodimer formation.***A*, schematic presentation of the RXRα homodimer formation assay. *B*, sGFP-RXRα LBD was titrated against 0.375 nM biotinylated RXRα LBD and 0.75 nM Tb-SA in presence of either 10 μM RXRα agonist SR11237 (*violet*) or no ligand at all (*blue*). *C*, the scheme shown in Figure 2*A* applies. sGFP-RXRα LBD was titrated against 0.375 nM biotinylated PPARγ LBD and 0.75 nM Tb-SA with either 10 μM RXRα agonist SR11237 (*cerise*) or no ligand (*light blue*). *B* and *C*, by addition of free sGFP the total concentration of sGFP was kept constant at 0.3 μM throughout all experiments. In the experiment on homodimer formation (*B*) with SR11237 the upper plateau is not reached. Therefore, the curve was fitted with the upper plateau from the apo experiment set as a fixed parameter. Data are the mean ± SD. R^2^ for each curve equals >98%. The experiments were all conducted three times (n = 3) with three technical replicates each (N = 3). One representative set of experiments each is shown that were conducted in parallel. The reported apparent *K*_*d*_ values (±SD) were calculated based on the entire data available, respectively. LBD, ligand-binding domain; PPAR, peroxisome proliferator-activated receptor; RXR, retinoid X receptor.

RXRα does not only function as an obligatory heterodimer partner for other nuclear receptors but can also form homodimers on its own. Therefore, we investigated the modulatory effect of the RXRα agonist SR11237 (BMS 649) on the stability of the RXRα homodimer. For this purpose we utilized the biotin-labeled RXRα LBD from the first series of experiments and the sGFP-RXRα from the second one (see Figs. 1*A*, 2*A*, and 3*A* for schematic representation of the assay setup).

In the experiment on homodimer formation (Fig. 3*B*) the upper plateau was not reached in the presence of 10 μM SR11237. However, the data from the apo experiment indicate that the upper plateau is reached once RXRα homodimer formation is fully achieved. In the experiment with SR11237 the compound was present at a constant concentration of 10 μM. No shift of the lower plateau was observed in comparison with the apo experiment. Hence, 10 μM SR11237 does not affect HTRF readout. The lower plateau results from diffusion-enhanced FRET, which in turn depends on the concentration of FRET donor and FRET acceptor, which are both kept constant throughout the entire experiment. The increase in HTRF upon complex formation depends on the particular complex formed and the concentration thereof. In this set of experiments the maximal concentration of FRET productive complex depends on the concentration of the FRET donor–coupled RXRα. These parameters were all identical in the apo experiment and the experiment with 10 μM SR11237. It is therefore reasonable to assume that also in presence of 10 μM SR11237 the curve would trend toward the upper plateau seen in the apo experiment. Henceforth, we utilized the upper plateau from the apo curve as a fixed parameter for fitting of the SR11237 data.

Of interest, binding of RXRα agonist SR11237 significantly destabilized the homodimer, which is reflected in an increase in the apparent *K*_*d*_ (*p* = 0.0083; 99% confidence interval [CI]; n = 3; N = 3 each; Fig. 3*B*).

For RXRα in complex with SR11237 (BMS 649) and a coactivator peptide two different crystal structures have been reported. Both show RXRα in agonist-bound active conformation, but the RXRα LBD is present either as an LBD monomer or as an LBD homodimer, respectively (11). This supports the assumption that SR11237 reduces the stability of the RXRα LBD homodimer under various experimental conditions.

Based on this observation, we then evaluated the modulatory effect of SR11237 on the formation of the PPARγ:RXRα heterodimer. In contrast to the destabilizing effect on the homodimer the RXRα agonist showed a significant stabilizing effect on the PPARγ:RXRα heterodimer as reflected in a reduction in the apparent *K*_*d*_ (*p* = 0.0007; 99% CI; n = 3; N = 3 each; Fig. 3*C*).

### PPARγ agonists promote recruitment of CBP-1 by isolated PPARγ LBD

The main function of nuclear receptors is the recruitment of transcription coregulators to specific DNA response elements. In *in vitro* assays this is reflected by the recruitment of small peptides that are derived from coactivators or negative regulators of transcription (corepressors).

In order to investigate coactivator recruitment by the isolated PPARγ LBD we coupled the biotin-labeled CBP-1 peptide to terbium-labeled streptavidin and detected the recruitment to the sGFP-PPARγ LBD in response to ligands. In this setup both FRET partners are present at a constant concentration, and hence, diffusion-enhanced FRET is not a matter of concern.

The optimal concentration of sGFP-PPARγ LBD for this assay type was evaluated in a separate control experiment (Fig. S7). When titrated in the presence of its reference agonist rosiglitazone a concentration of 100 nM sGFP-PPARγ resulted in a substantial gain in FRET. At this concentration the apo titration series did still show almost no increase in comparison with the baseline in HTRF detected with lower sGFP-PPARγ concentrations or no protein at all. Therefore, 100 nM was chosen as the default LBD concentration in all further LBD monomer cofactor recruitment assays.

**Figure 4:**
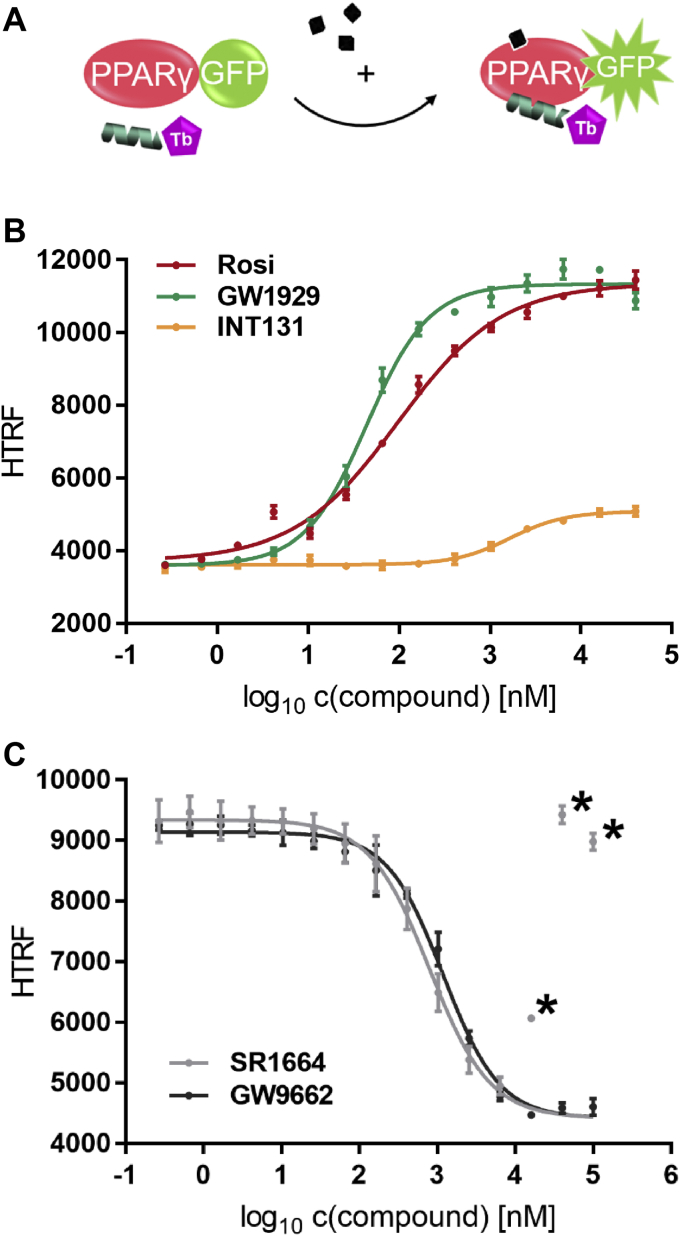
**Ligand-dependent recruitment of CBP-1 by PPARγ LBD.***A*, schematic representation of the PPARγ monomer cofactor recruitment assay. *B*, PPARγ full agonist GW1929 (*green*), partial agonist INT131 (*yellow*), and reference agonist rosiglitazone (*red*) were titrated against 100 nM sGFP-PPARγ LBD, 12 nM Tb-SA, and 12 nM biotinylated CBP-1 cofactor peptide. *C*, PPARγ irreversible antagonist GW9662 (*black*) and nonagonist SR1664 (*gray*) were titrated against 12 nM Tb-SA, 12 nM biotinylated CBP-1 cofactor peptide, and 100 nM sGFP-PPARγ LBD activated with uniformly 1 μM rosiglitazone. In both experiments homogeneous time-resolved FRET measurements were performed after 1 h incubation at room temperature. Data are the mean ± SD; N = 3. R^2^ for Rosi and GW1929 equals >99%, for INT131 it equals 96.8%. With SR1664 the lower plateau is not reached and the three highest concentrations tested resulted in unspecific aggregation. Asterisks indicate these data points that were identified as outliers using GraphPad with Q, the desired maximum false discovery rate, being set to 1%. The curve for SR1664 was fitted with the lower plateau from the GW9662 curve set as a fixed parameter. R^2^for GW9662 equals 98.9%. CBP-1, CREB-binding protein coactivator motif 1; LBD, ligand-binding domain; PPAR, peroxisome proliferator-activated receptor; Tb-SA, terbium cryptate conjugated to streptavidin.

Rosiglitazone activated CBP-1 recruitment with an EC_50_ of 91 nM, and its maximal efficacy was set to 100%. GW1929 promoted recruitment with an EC_50_ of 44 nM and comparable efficacy. The partial agonist INT131 mediates less cofactor recruitment, which is indicated by a higher EC_50_ and impaired maximal efficacy of 19% compared with rosiglitazone (*p* < 0.0021 for efficacy and *p* < 0.0001 for EC_50_ in sum-of-squares F-test; Fig. 4*B*, Table 1).

**Table 1:** Overview of potency and efficacy of all PPARγ ligands tested for modulation of CBP-1 recruitment by the PPARγ LBD either on isolated PPARγ LBD (monomer recruitment) or in the context of PPARγ LBD in complex with RXRα LBD (dimer recruitment)

Ligand	Monomer recruitment		Dimer recruitment	
	Agonist mode			
	Potency (EC_50_)	Efficacy (%)	Potency (EC_50_)	Efficacy (%)
Rosi	91 ± 8 nM^a^	100	94 ± 3 nM^a^	100
GW1929	44 ± 3 nM	101	87 ± 7 nM	118
INT131	1.7 ± 0.2 μM	19	1.2 ± 4 μM	26

	Antagonist mode *versus* 1 μM Rosi			
	Potency (IC_50_)	Max inhibition (%)	Potency (IC_50_)	Max inhibition (%)
SR1664	0.8 ± 0.06 μM	65	1.5 ± 0.2 μM	70
GW9662	1.2 ± 0.07 μM	62	12 ± 2 μM	52

Normalized to rosiglitazone separately for recruitment to the isolated PPARγ LBD or in the context of the heterodimer with the RXRα LBD mutant incapable of recruiting coactivators on its own. EC_50_ and IC_50_ values are reported with SD.

a In order to provide information on reproducibility and robustness of the assays, the experiments with reference agonist rosiglitazone were repeated in four independent experiments (n = 4; each with N = 3). For all other ligands, experiments were conducted once with three technical replicates (N = 3). As a control a dilution series with rosiglitazone was always conducted in parallel.

In the antagonist mode of the cofactor recruitment assay PPARγ is constantly activated by 1 μM rosiglitazone, which corresponds to approx. EC_80_. These conditions were used to investigate the known antagonists SR1664 and GW9662 for their potential to impede activation by rosiglitazone and, hence, reverse CBP-1 recruitment. Both antagonists were able to fully inhibit coactivator recruitment. GW9662 showed an IC_50_ of 1.2 μM, and SR1664 of 0.8 μM (Fig. 4*C*, Table 1).

### RXRα agonist SR11237 does not affect cofactor recruitment by the isolated PPARγ LBD

**Figure 5:**
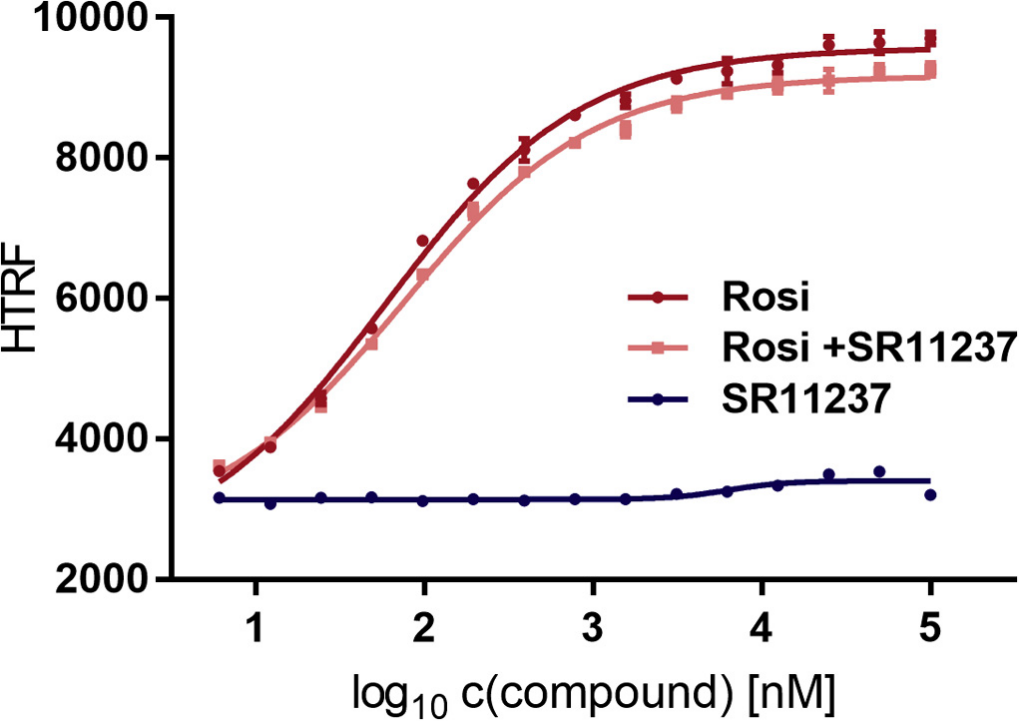
**Effect of RXRα agonist SR11237 on PPARγ LBD.** The scheme shown in Figure 4*A* applies. RXRα agonist SR11237 (*blue*) or PPARγ reference agonist rosiglitazone (*red*) was titrated against 100 nM sGFP-PPARγ LBD, 12 nM Tb-SA and 12 nM biotinylated CBP-1 cofactor peptide. In a third experiment the modulatory potential of titrated rosiglitazone was challenged with constant 10 μM SR11237 (*salmon*). Data are the mean ± SD; N = 3. R^2^ for the fit curve for SR11237 alone equals 91%. R^2^ for Rosi alone or in combination with SR11237 equals ≥99.5%, respectively. CBP-1, CREB-binding protein coactivator motif 1; LBD, ligand-binding domain; PPAR, peroxisome proliferator-activated receptor; RXR, retinoid X receptor; Tb-SA, terbium cryptate conjugated to streptavidin.

Next, we intended to determine whether the increased heterodimer formation in response to incubation with RXRα agonist SR11237 can be explained exclusively with selective binding to RXRα. Therefore, we probed the effect of SR11237 on PPARγ in the monomer cofactor recruitment assay (scheme as in Fig. 4*A*). As shown in Figure 5, SR11237 neither activated recruitment of CBP-1 by PPARγ nor impaired rosiglitazone-mediated recruitment.

### Design of a recruitment-incapable RXRα mutant

In this study, we aimed to investigate PPARγ coactivator recruitment in the context of the LBD:LBD heterodimer with RXRα. As both nuclear receptors are able to recruit cofactors a recruitment-incapable RXRα LBD mutant had to be designed.

**Figure 6:**
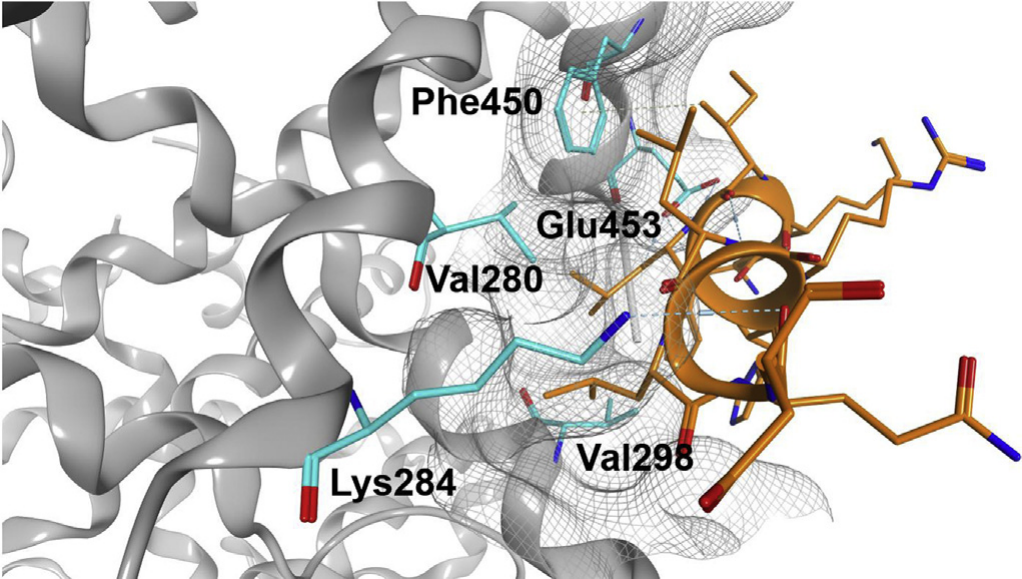
**RXRα wildtype AF-2 with bound SRC1 cofactor peptide.** The graphic shows the SRC1 cofactor peptide (*orange*) bound to the AF-2 of wildtype RXRα LBD. The five RXRα residues that are mutated in the mutant construct are highlighted in *cyan*. Val280, Val298, and Phe450 contribute to the hydrophobic cleft accommodating the leucine side chains of the coactivator LXXLL consensus motif while Lys284 and Glu453 form a charge clamp stabilizing the dipole of the coactivator helix (Protein Data Bank: 3r5m). AF-2, activation function 2; LBD, ligand-binding domain; RXR, retinoid X receptor; SRC1, steroid receptor coactivator 1.

Hence, we analyzed the AF-2 coactivator-binding site (ligand-dependent activation function 2) in the structure of agonist-bound RXRα published by Zhang *et al.*; Figure 6 (12). The residues of the coactivator consensus motif LXXLL bind to a hydrophobic groove. Residues Val280, Val298, and Phe450 contribute to the hydrophobic surface of this groove and form van-der-Waals contacts with the leucine residues of the coactivator-derived peptide. The binding groove is flanked by Lys284 and Glu453 that form a charge clamp stabilizing the dipole of the coactivator helix (13).

The hydrophobic residues were mutated to Thr in case of Val 280 and 298, or Tyr in case of Phe 450. In this way the mutations were rather conservative with only one additional –OH being introduced each. This should disrupt the hydrophobic area. Furthermore, we investigated the effect of introducing charge clamp mutations Lys284Glu and Glu453Arg, which should invert the distribution of the most important charges that flank the coactivator-binding groove.

The following mutants were generated: the VVF mutant (V280T + V298T + F450Y); two single mutants E453R and K284E, as well as the total mutant harboring all five exchanges.

**Figure 7:**
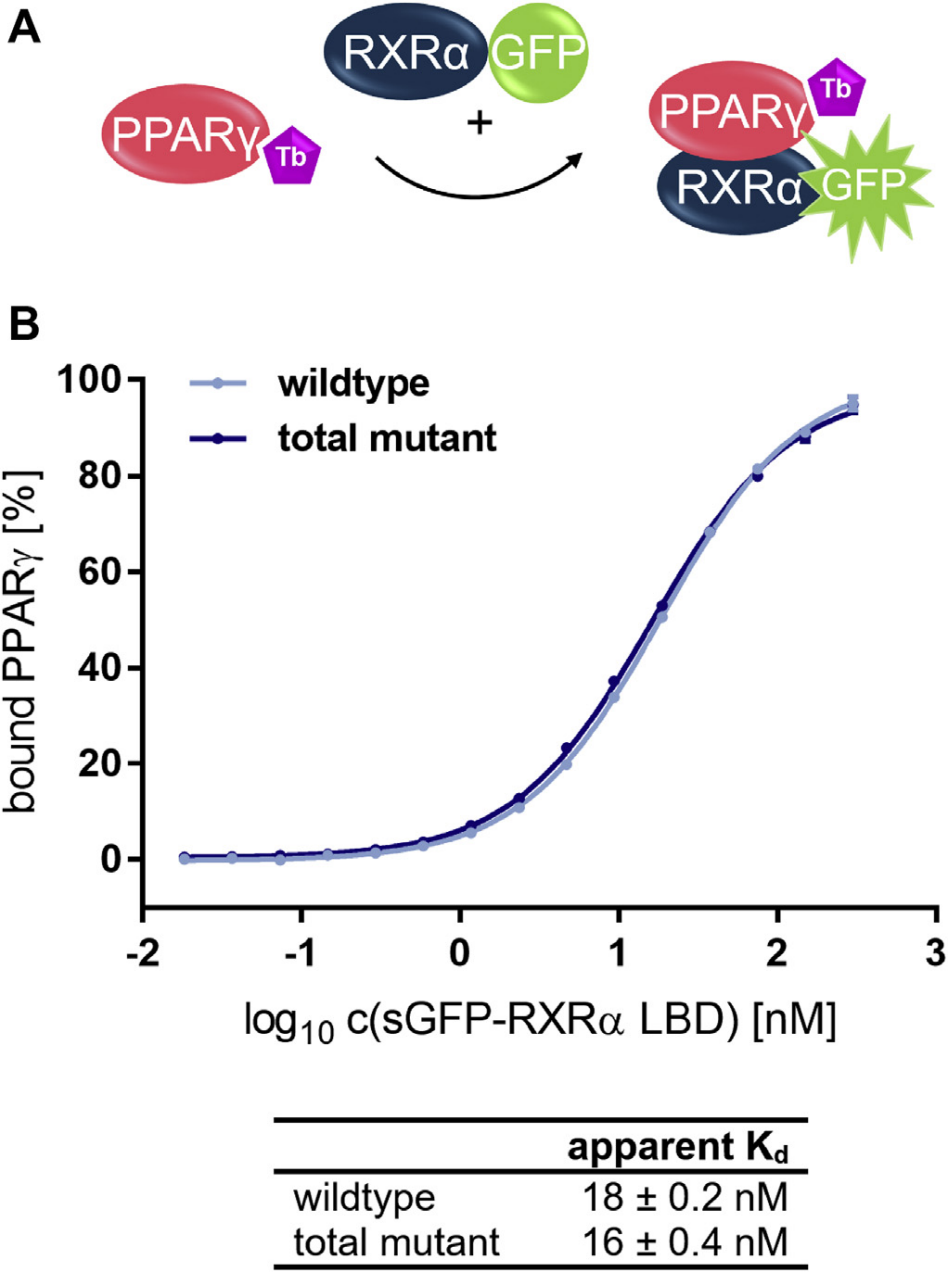
**Formation of the LBD:LBD heterodimer between PPARγ and mutant RXRα in comparison with wildtype.***A*, schematic representation of the heterodimer formation assay with PPARγ LBD being coupled to the FRET donor. *B*, sGFP-RXRα LBD wildtype (*light blue*) or mutant (*dark blue*) was titrated against 0.375 nM biotinylated PPARγ LBD and 0.75 nM Tb-SA. By addition of free sGFP the total concentration of sGFP was kept constant at 0.3 μM throughout the entire experiment. The *curves* show similar dimerization behavior for RXRα total mutant (V280T, K284E, V298T, F450Y, and E453R) in comparison with the wildtype RXRα LBD. Data are the mean ± SD; N = 4. R^2^ for each curve equals >99%. LBD, ligand-binding domain; PPAR, peroxisome proliferator-activated receptor; RXR, retinoid X receptor; sGFP, super folder green fluorescent protein; Tb-SA, terbium cryptate conjugated to streptavidin.

All mutants were examined for their interaction with PPARγ LBD (Figure 7; Fig. S8).

The total mutant differed least from wt RXRα in terms of the apparent *K*_*d*_ for heterodimer formation. Since such unchanged interaction with PPARγ was the most important criterion for use in later assay setups, the total mutant was selected and further characterized.

A modified Gal4 transactivation assay was used to assess RXRα wt and RXRα mutant in their ability to recruit PPARγ LBD in a cellular context. A schematic depiction of this assay is shown in Fig. S9. RXRα LBD is expressed as a fusion protein with Gal4 DNA-binding domain (DBD) that guides RXRα to the Gal4 DNA response elements in the promoter region of the firefly reporter gene. The PPARγ LBD is expressed as a fusion protein with VP16, a strong trans-inducer of transcription (14). Formation of the RXRα:PPARγ heterodimer results in recruitment of VP16 to the Gal4 response elements and subsequently activates expression of firefly luciferase (Luc).

**Figure 8:**
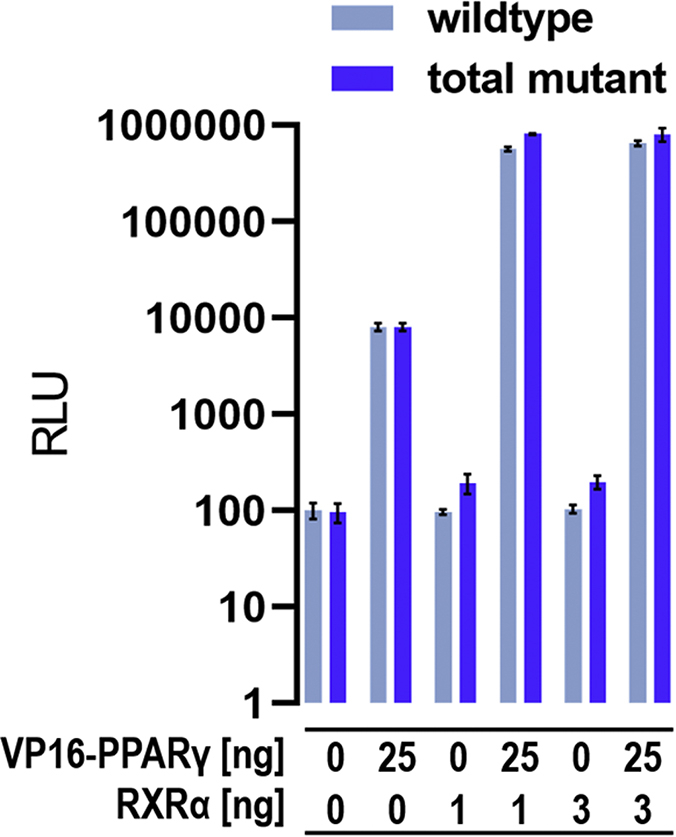
**Validation of RXRα wildtype and total mutant heterodimer formation with PPARγ in a cellular setting.** HEK293T cells were cotransfected with 1 or 3 ng of either Gal4-RXRα wildtype or Gal4-RXRα total mutant plasmid, and/or 25 ng VP16-PPARγ plasmid, in any case in combination with the plasmids for firefly reporter and Renilla luciferase. Control experiments without any nuclear receptor plasmid and with VP16-PPARγ alone were conducted for both experiment series (wt and mutant), respectively. Luciferase fluorescence was detected using the Dual-Glo Luciferase Assay System (Promega). Data are the mean ± SD; n = 5. PPAR, peroxisome proliferator-activated receptor; RXR, retinoid X receptor.

HEK293T cells were transfected with plasmids for the firefly Luc reporter and constitutively expressed Renilla Luc, which served as an internal control. Cotransfection of either Gal4-RXRα wildtype (wt) or Gal4-RXRα mutant alone did not enhance expression of firefly Luc (Fig. 8). When only VP16-PPARγ was cotransfected, activity of firefly Luc was enhanced approximately 100-fold. The combination of VP16-PPARγ and Gal4-RXRα resulted in an additional approximately 100-fold increase. This was observed for both RXRα wt and the RXRα mutant and showed that the introduced mutations do not alter dimer formation between RXRα and PPARγ.

### RXRα mutant is incapable of recruiting SRC1

**Figure 9:**
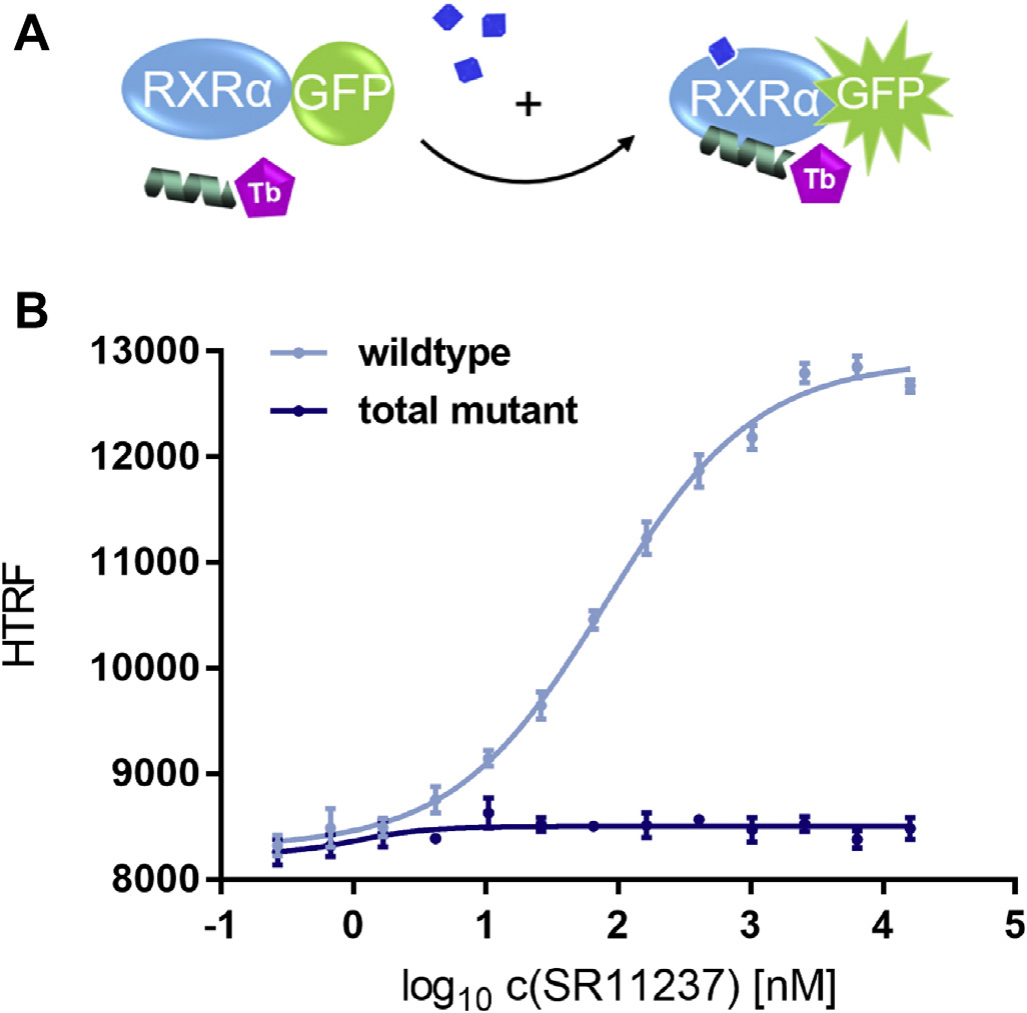
**Validation of block of coactivator recruitment by mutations introduced into RXRα LBD.***A*, schematic representation of cofactor recruitment assay employing isolated RXRα LBD and coactivator peptide coupled to the FRET donor. *B*, RXRα reference agonist SR11237 was titrated to 100 nM sGFP-RXRα LBD wildtype (*light blue*) or total mutant (*dark blue*), 12 nM Tb-SA, and 12 nM biotinylated SRC1-2 cofactor peptide. For the mutant no stimulation of coactivator recruitment was observed. Data are the mean ± SD; N = 4. R^2^ for each curve equals >99%. LBD, ligand-binding domain; RXR, retinoid X receptor; sGFP, super folder green fluorescent protein; SRC1-2, steroid receptor coactivator 1 motif 2; Tb-SA, terbium cryptate conjugated to streptavidin.

We used the monomer coactivator recruitment assay to validate that the introduced mutations indeed prevent coactivator recruitment by RXRα in response to SR11237 (Fig. 9).

**Figure 10:**
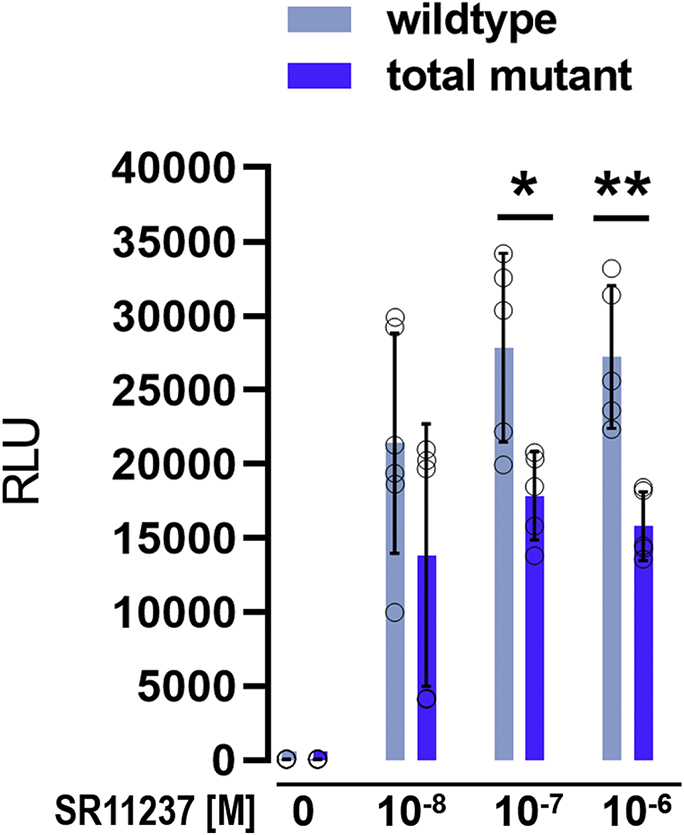
**Transactivation mediated by Gal4-RXRα in response to SR11237.** HEK293T cells were cotransfected with 0.3 ng of either Gal4-RXRα wildtype or Gal4-RXRα total mutant plasmid, and 1 ng of the VP16-SRC1-2 plasmid, always in combination with the plasmids for firefly reporter and Renilla luciferase. Cells were stimulated with SR11237 in medium containing 0.1% dimethyl sulfoxide or 0.1% dimethyl sulfoxide alone. Luciferase fluorescence was detected using the Dual-Glo Luciferase Assay System (Promega). Data are the mean ± SD; n = 5. ∗*p* < 0.05; ∗∗*p* < 0.005. RLU, relative light unit; RXR, retinoid X receptor; SRC1-2, steroid receptor coactivator 1 motif 2.

To further validate this, we employed another variation of the cell-based Gal4 transactivation assay. When activated by one of its ligands, Gal4-RXRα wt can recruit components of the transcription machinery thereby promoting transcription of the reporter gene (firefly Luc). But RXRα LBD is also an obligate heterodimer partner for various other NRs. Henceforth, ligand-dependent activation of RXRα could in principle not only cause recruitment of coactivators but also modulate various other interactions that influence transactivation. In order to intensify the gain in transactivation that is directly related to the recruitment of a coactivator we, therefore, cotransfected a plasmid for expression of VP16 coupled to SRC1 coactivator motif 2. Control experiments showed that, in the absence of an RXRα agonist, coexpression of Gal4-RXRα does not promote transactivation in comparison with cells expressing only VP16-SRC1 (Fig. S10). Stimulation with SR11237 in the same setting resulted in a substantial increase in transactivation of reporter gene expression. And at ≥ 0.1 μM SR11237 transactivation by the RXRα mutant was markedly reduced in comparison with RXRα wt (Fig. 10).

This provided further evidence that the mutations prevent coactivator recruitment. However, also on cells transfected with the RXRα mutant stimulation with SR11237 resulted in a substantial increase in transactivation compared with dimethyl sulfoxide (DMSO) control. This indicates that the mutations do not prevent binding of SR11237.

In order to reassure that the inability of the RXRα total mutant LBD to recruit the SRC1 cofactor peptide is not caused by a defect in ligand binding, direct binding of the reference agonist SR11237 was examined using isothermal titration calorimetry. The experiments confirmed that both the wildtype as well as the total mutant LBD bind SR11237 with comparable affinity (Figs. S11 and S12).

### Coactivator recruitment by PPARγ LBD can be assayed in the context of the heterodimer with RXRα

Utilizing the RXRα LBD total mutant we set up an assay to specifically trace the coactivator recruitment of PPARγ LBD in the context of the LBD:LBD heterodimer. Based on the *K*_*d*_ values observed in previous experiments (Figs. 2 and 3), a fixed concentration of 2 μM sGFP-coupled RXRα LBD total mutant was deemed sufficient to ensure that under all conditions investigated at least 90% of PPARγ LBD is bound in the heterodimer with RXRα.

**Figure 11:**
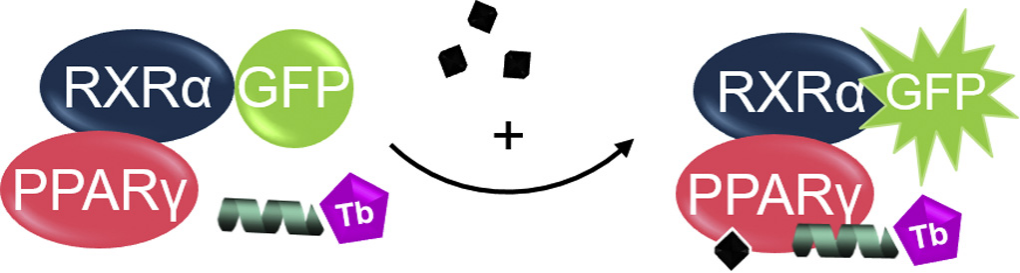
**Schematic representation of the heterodimer coactivator recruitment assay.** Presence of PPARγ LBD and a 20-fold molar excess of sGFP-RXRα LBD results in the major fraction of PPARγ being incorporated in PPARγ:RXRα heterodimers. The employed RXRα LBD is incapable of recruiting coactivators on its own; mutations V280T, K284E, V298T, F450Y, and E453R (total mutant). A biotin-labeled coactivator peptide is coupled to Tb-SA. Recruitment of the coactivator peptide by PPARγ in complex with sGFP-RXRα results in FRET between Tb and sGFP. LBD, ligand-binding domain; PPAR, peroxisome proliferator-activated receptor; RXR, retinoid X receptor; sGFP, super folder green fluorescent protein; Tb-SA, terbium cryptate conjugated to streptavidin.

In contrast to the monomer recruitment assay, PPARγ now lacks the sGFP-tag, and hence, only the fraction incorporated in the heterodimer can produce a HTRF signal upon coactivator binding; scheme in Figure 11.

In comparison with coactivator recruitment by the isolated monomeric PPARγ LBD we found in the heterodimer setting that the EC_50_ observed for GW1929 increased by about 2-fold, whereas the EC_50_ values of rosiglitazone and INT131 were unaffected. The IC_50_ values observed for the PPARγ antagonists SR1664 and GW9662 when tested against 1 μM rosiglitazone (approximately EC_80_) were 2- to 4-fold higher in the context of the heterodimer (Table 1; Fig. S13).

### Complexation of PPARγ LBD into the heterodimer with RXRα causes an increase in its basal affinity for the coactivator CBP-1

Furthermore, we observed that, in comparison with the monomer coactivator recruitment assay the assay window happened to be much smaller when testing PPARγ activation in the context of the heterodimer with RXRα as shown in Fig. S14 for the activation with rosiglitazone.

**Figure 12:**
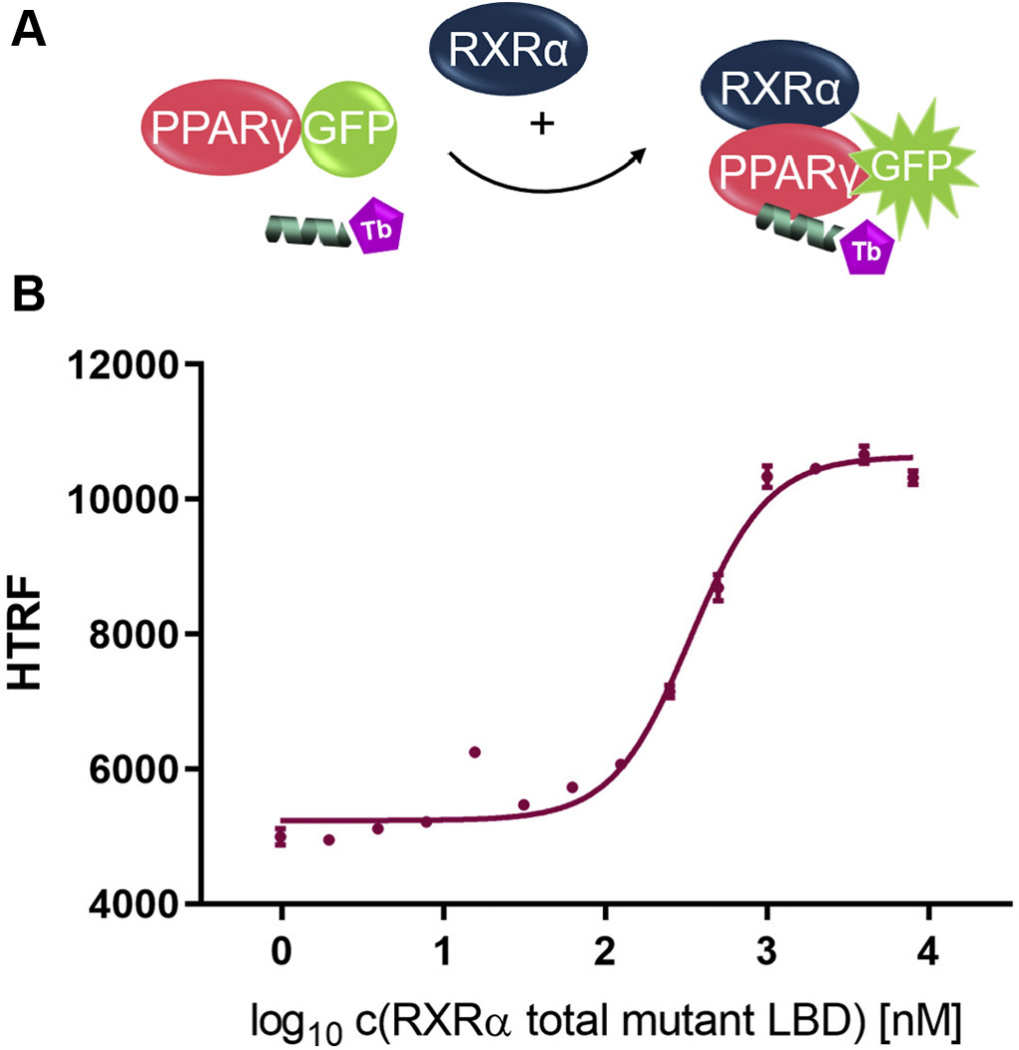
**Enhanced basal affinity between apo PPARγ LBD and coactivator CBP-1 through incorporation of PPARγ into the LBD:LBD heterodimer with RXRα.***A*, schematic representation of the assay setup. *B*, RXRα total mutant LBD was titrated against 100 nM sGFP-PPARγ LBD, 12 nM Tb-SA, and 12 nM biotinylated CBP-1 cofactor peptide. Data are the mean ± SD; N = 3. R^2^ equals 98.7%. CBP-1, CREB-binding protein coactivator motif 1; LBD, ligand-binding domain; PPAR, peroxisome proliferator-activated receptor; RXR, retinoid X receptor; sGFP, super folder green fluorescent protein; Tb-SA, terbium cryptate conjugated to streptavidin.

Therefore, we investigated to which extent the formation of the dimer affects basal coactivator recruitment by PPARγ. As in the monomer recruitment assay, we observed CBP-1 recruitment by PPARγ coupled to sGFP, but titrated free RXRα total mutant LBD instead of ligand (Fig. 12). Upon formation of the heterodimer with RXRα the basal affinity of PPARγ for the CBP-1 peptide increased substantially.

### In the heterodimer the RXRα agonist SR11237 moderates activation of PPARγ by rosiglitazone

**Figure 13:**
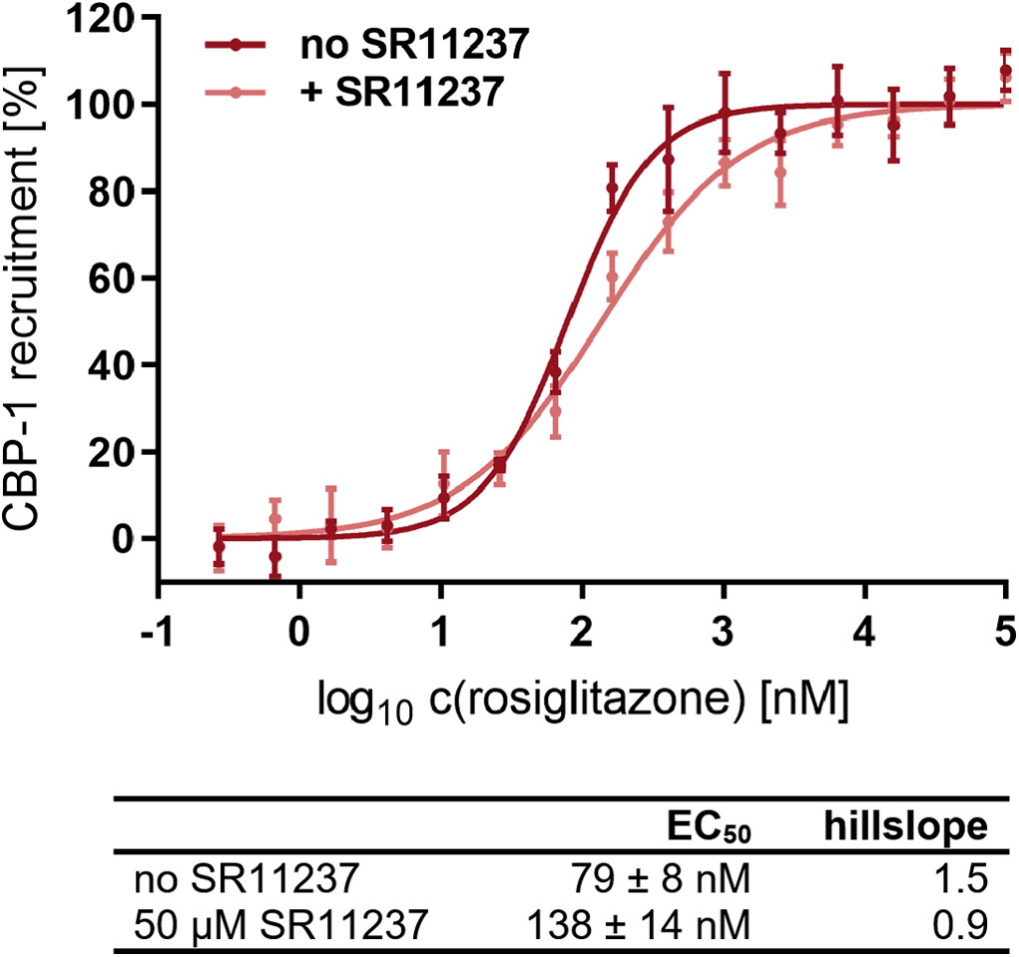
**In the heterodimer with RXRα CBP-1 recruitment by PPARγ in response to rosiglitazone is moderated by RXRα agonist SR11237.** The scheme shown in Figure 11 applies. One-hundred nanomolar label-free PPARγ LBD incorporated into the heterodimer with sGFP-RXRα (total mutant; 2 μM) was stimulated with rosiglitazone. Recruitment of biotinylated CBP-1 cofactor peptide (12 nM) coupled to Tb-SA (12 nM) was detected by homogeneous time-resolved FRET after 1 h incubation with or without 50 μM SR11237. Basal and upper plateaus were comparable between both experiments, and curves were fitted to a scale of 0% to 100% of observed gain in CBP-1 recruitment. One representative experiment out of three independent repeats (n = 3) is shown. The reported EC_50_ values refer to this experiment, respectively. Data are the mean ± SD; N = 3. R^2^ for each curve equals >99%. CBP-1, CREB-binding protein coactivator motif 1; LBD, ligand-binding domain; PPAR, peroxisome proliferator-activated receptor; RXR, retinoid X receptor; sGFP, super folder green fluorescent protein; Tb-SA, terbium cryptate conjugated to streptavidin.

Next we investigated whether activation of RXRα by SR11237 modulates coactivator recruitment by PPARγ in response to activation by rosiglitazone. We used 50 μM SR11237 to ensure saturation of RXRα (2 μM) in this setting. Basal and upper plateaus were comparable between both experiments, with the latter indicating maximal activation of PPARγ to be reached. Therefore, the curves were fitted to a scale of 0% to 100% of observed gain in CBP-1 recruitment. The dose–response curves revealed that EC_50_ and hillslope are substantially altered in the presence of SR11237 (*p* < 0.0108 in sum-of-squares F-test; Fig. 13). Occupation of RXRα with SR11237 resulted in a significant reduction of the hillslope (*p* = 0.012; 95% CI; n = 3). This indicates a change in cooperativity of the conformational changes necessary to accommodate rosiglitazone and the CBP-1 peptide. The SR11237-provoked increase in EC_50_ varied when the experiments were conducted several times (n = 3). This technically resulted in a low degree of assuredness for the claim that SR11237 increases the EC_50_ (*p* = 0.27). The EC_50_ in presence of SR11237 was, however, for all repetitions determined to be 138 nM or higher (mean: 138, 271, 886 nM). As the EC_50_ in absence of SR11237 was not equally affected by statistical variations (mean: 75–105 nM), this puts the suggestion close, that SR11237 indeed hinders activation of PPARγ by rosiglitazone.

We previously demonstrated that binding of SR11237 to RXRα destabilizes the RXRα wildtype homodimer (Fig. 3*B*). As the heterodimer recruitment assay utilizes RXRα total mutant we investigated to which degree SR11237 also reduces its tendency to form homodimers (Fig. S15). The observation most important for the further experiments was that treatment with 10 μM SR11237 increased the apparent *K*_*d*_ of homodimer formation to about 150 to 200 nM for both wildtype and RXRα total mutant.

### SR11237 can indirectly activate PPARγ coactivator recruitment owing to increased formation of the LBD heterodimer with RXRα

**Figure 14:**
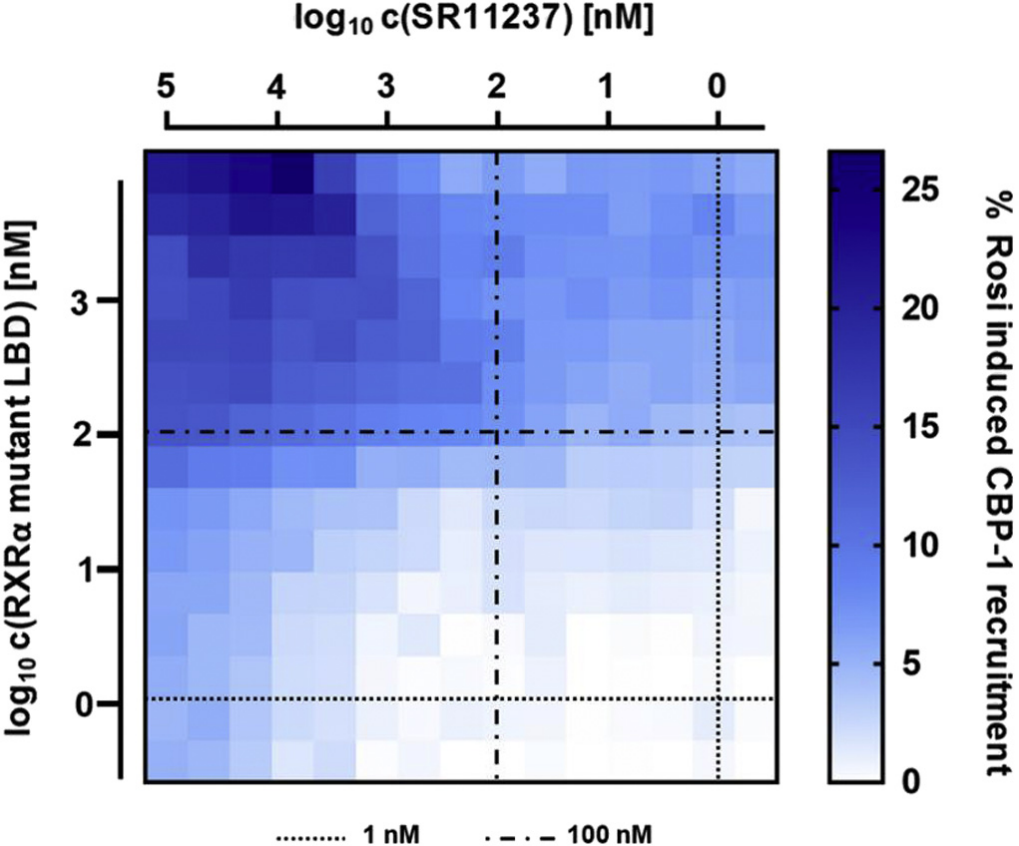
**Cooperative effect of increasing concentrations of mutant RXRα LBD and SR11237 on promoting recruitment of coactivator CBP-1 by the PPARγ LBD.** One-hundred nanomolar sGFP-PPARγ LBD was used throughout. Recruitment of biotin-labeled CBP-1 coactivator peptide (12 nM) coupled to Tb-SA (12 nM) was detected as an increase in homogeneous time-resolved FRET. The recruitment was stimulated by titration of SR11237 (up to 100 μM) and/or unlabeled mutant RXRα LBD (up to 8 μM; mutations blocking cofactor recruitment) and referenced to activation of isolated sGFP-PPARγ LBD by its agonist rosiglitazone. Data are the mean; N = 2 (SD is reported separately in the Supplemental Data). CBP-1, CREB-binding protein coactivator motif 1; LBD, ligand-binding domain; PPAR, peroxisome proliferator-activated receptor; RXR, retinoid X receptor; sGFP, super folder green fluorescent protein; Tb-SA, terbium cryptate conjugated to streptavidin.

Next we investigated if this destabilization of the RXRα homodimer by SR11237 is capable of indirectly stimulating CBP-1 recruitment by PPARγ as a result of enhanced incorporation of PPARγ into the heterodimer. For this purpose, we changed the experimental setting back to the monomer recruitment assay utilizing sGFP-PPARγ LBD and titrated unlabeled recruitment-incapable RXRα LBD (total mutant) and/or SR11237 (Fig. 14). CBP-1 recruitment by isolated sGFP-PPARγ upon stimulation with rosiglitazone was used as benchmark for grading of PPARγ activation. When the other factor was present at ≤1 nM, and hence, at a concentration far below equimolar to PPARγ (100 nM), neither SR11237 nor RXRα was capable of provoking more than 5% of CBP-1 recruitment seen with rosiglitazone. However, at concentrations equal to that of PPARγ and higher, combined addition of SR11237 and RXRα showed a strong synergistic effect with up to 26% of CBP-1 recruitment relative to full activation of monomeric PPARγ with rosiglitazone.

Of interest, this gain in coactivator recruitment is close to the degree of activation of PPARγ reported for various partial agonists such as INT131 (30%) (15) or SR145 and SR147 (each ~35%) (16) as determined in HTRF-based *in vitro* binding assays and compared with rosiglitazone, respectively. On monomeric PPARγ in our assays INT131 resulted in up to 19% normalized CBP-1 recruitment. In the context of the heterodimer with RXRα this increased to 26% (Table 1).

### Tetrac is a natural PPARγ agonist and promotes formation of the heterodimer with RXRα

We recently identified classical and nonclassical thyroid hormones to directly activate PPARγ and RXRα. L-thyroxin (T4) can be metabolized in various ways. The product of its oxidative deamination is the nonclassical thyroid hormone 3,3',5,5'-tetraiodothyroacetic acid (TETRAC). Among the thyroid hormones TETRAC was the most active on PPARγ and also showed activity on all three RXRs (17).

Depending on the coactivator assayed (CBP or SRC1) stimulation of PPARγ with TETRAC resulted in 27% to 37% of the gain in coactivator recruitment mediated by 1 μM rosiglitazone (~EC_80_). Activation of RXRα was below 10% in comparison with 1 μM SR11237. Hence, separately on both receptors TETRAC acted as a partial agonist. However, in the heterodimer coactivator recruitment assay TETRAC had a much stronger influence on CBP-1 recruitment by the PPARγ LBD. In comparison with 1 μM rosiglitazone, recruitment was enhanced by approximately 50%, which qualifies TETRAC as a full PPARγ agonist particularly in the context of the PPARγ:RXRα heterodimer (17).

**Figure 15:**
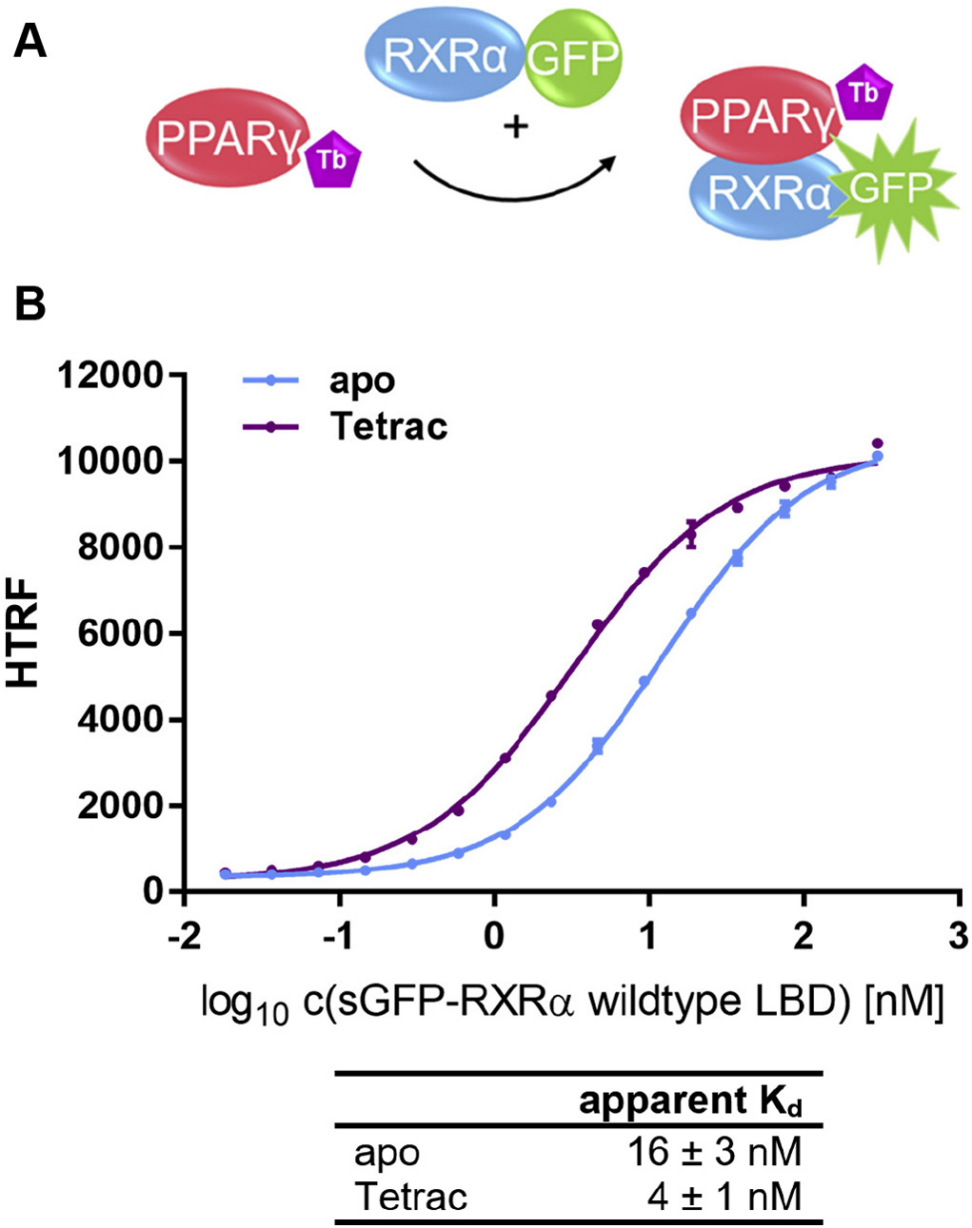
**Tetrac modulates formation of the LBD:LBD heterodimer between PPARγ and RXRα (wt).***A*, schematic representation of the heterodimer formation assay with PPARγ LBD being coupled to the FRET donor and sGFP-RXRα wildtype being titrated. *B*, sGFP-RXRα LBD wildtype was titrated against 0.375 nM biotinylated PPARγ LBD and 0.75 nM Tb-SA. By addition of free sGFP the total concentration of sGFP was kept constant at 0.3 μM throughout the entire experiment. Treatment with 10 μM Tetrac resulted in a 4-fold reduction in the apparent *K*_*d*_ of dimer formation in comparison with dimethyl sulfoxide control. Data are the mean ± SD; N = 3. The experiments were conducted three times (n = 3) with three technical replicates each (N = 3). One representative set of experiment each is shown. R^2^ for each curve equals >99%. The reported apparent *K*_*d*_ values (±SD) were calculated based on the entire data available, respectively. LBD, ligand-binding domain; PPAR, peroxisome proliferator-activated receptor; RXR, retinoid X receptor; sGFP, super folder green fluorescent protein; Tb-SA, terbium cryptate conjugated to streptavidin.

We therefore questioned, to which extent TETRAC may modulate the oligomeric state of the RXRα homodimer (Fig. S16) or the PPARγ:RXRα heterodimer (Fig. 15).

The formation of the RXRα homodimer was slightly but significantly weakened in presence of 10 μM TETRAC (*p* = 0.0125; 95% CI; n = 3). This was expected as the TETRAC-mediated activation of RXRα had been weak and also associated with a rather high EC_50_ of approximately 10 μM (17).

The PPARγ:RXRα heterodimer was much more affected. TETRAC strongly reduced the apparent *K*_*d*_ of formation of the PPARγ:RXRα (wt) heterodimer significantly (*p* = 0.0237; 95% CI; n = 3). This makes TETRAC the PPARγ agonist with the strongest heterodimer-stabilizing effect among all agonists tested in this study. Moreover, the observed gain in stability of the heterodimer is coherent with the substantial gain in TETRAC-activated coactivator recruitment seen in PPARγ accompanied by RXRα in comparison with PPARγ alone.

## Discussion

Nuclear receptors generally function as homo- or heterodimers (with the exception of type IV receptors that bind DNA as monomers) (5). In a two-step process, dimerization is first initiated through the LBD dimerization interface leading to the formation of the dimer in solution (18). Formation of the second dimer interface between DBDs and LBDs of the partnering NRs then defines the relative positions of the DBDs, which in turn restricts receptor binding to their cognate DNA response elements (19).

The fact that receptor dimerization precedes DNA binding and that aside PPARγ various other NRs compete for the same heterodimer partners would in principle dictate to study ligand-dependent cofactor recruitment alongside LBD heterodimer formation.

However, as a consequence of technical limitations the currently applied methods neglect the process of NR dimer formation prior to DNA binding. NRs have preferences for different coactivators that share the common consensus motive LXXLL. Consequently, many coactivators can be recruited by various NRs, and this in turn impedes that coactivator recruitment could be traced back to a particular NR in the cellular environment. Henceforth, transactivation assays with full-length NRs rely on overexpression of the target NR in combination with reporter plasmids that harbor DNA response elements specific to the particular NR. Interpretation of these assays is often complicated owing to secondary effects on cell metabolism. Therefore, the current gold standard for evaluation of NR modulators is either ligand-dependent cofactor recruitment to the isolated LBD *in vitro* or cell-based assays that employ fusion proteins of the NR LBD and Gal4 DBD (20). Although these assays offer reliable detection of coactivator recruitment, they lack the opportunity to study LBD dimerization and how it influences coactivator recruitment. Consequently, the modulation of dimer formation upon ligand binding has not been investigated in detail yet, and also the interplay of dimer formation and cofactor recruitment remains underexplored.

Thus, an improved holistic understanding could provide new possibilities for ligand development in the field of NR ligands. The complexity of these processes requires a toolkit that allows one to study cofactor recruitment in the context of NR heterodimers and how it is interconnected with LBD oligomerization equilibria.

The two-dimensional titration experiment exemplifies the opportunities that the newly designed assay setup provides for the study of this complex cross talk (Fig. 14). SR11237 neither activates nor inhibits PPARγ. However, it destabilizes the RXRα homodimer, whereas it enhances the affinity of RXRα to form heterodimers with PPARγ. Upon SR11237 addition RXRα is set free from the homodimer and is consequently available for formation of the heterodimer with PPARγ. In response to SR11237, RXRα might generally be more available for formation of heterodimers, more precisely those heterodimers that can accommodate the RXR ligand without a decrease in stability of the heterodimer.

Several studies have shown the importance of enhanced understanding of NR cross talk. Wang *et al.* (21) observed on the heterodimer composed of the LBDs of RXRα and FXR (farnesoid X receptor) that in the absence of ligands both receptors show higher affinity for SRC1 when they are part of the heterodimer than in comparison with the monomeric LBD, respectively. FXR agonists and the RXRα agonist 9-*cis*-RA synergistically enhance SRC1 recruitment. Structural comparison of the FXR monomer and the FXR:RXRα heterodimer indicates that conformational changes are induced by both formation of the heterodimer and ligand binding (21). In a two-hybrid reporter gene assay it had been shown that the ligand-dependent activation function AF-2 of RXRα is not required for RXRα- and PPARγ-specific ligands to activate PPARγ individually or synergistically. But on the contrary, mutation of PPARγ AF-2 prevented the RXRα:PPARγ heterodimer to respond to ligands specific to any of the LBDs (22). In both receptors the authors had introduced mutations into helix 12 that block its interaction with the core of the LBD and thereby prevent formation of the AF-2, respectively. However, based on their data we assume that in effect this may be comparable with a simple truncation of helix 12 and, hence, prone to affect allosteric cross talk, not only within the LBD but also across the dimerization interface. Consequently, this prohibited the use of the same mutation strategy for the development of our assays.

Usually recruitment of coactivators is ligand dependent, whereas corepressors in most cases happen to interact with unliganded (apo) receptors. And hence, the regulation of coactivator recruitment is in general of higher importance in drug development for nuclear receptors. For this reason, our assay strategy focused on coactivator recruitment.

A special construct used in this study is the mutated RXRα LBD, which is incapable of binding a coactivator peptide. Using statistical coupling analysis (SCA) to detect coevolution of amino acids in a protein, a network of 27 energetically coupled residues has been identified that mediates allosteric signaling in RXRα heterodimers (23). The recruitment-deficient RXRα LBD mutant characterized and utilized in this publication encompasses two mutations of residues assigned by SCA (Phe450 and Lys284). Phe450 is not located within physical contact distance to any other cluster position within RXRα but instead forms contacts only to the LXXLL coactivator motif. Hence, with regard to the network of signal transducing residues within RXRα Phe450 is isolated in the absence of a coactivator peptide. Lys284, which was mutated to Glu, is within contact distance to other residues assigned by SCA also from within the RXRα LBD. However, its most important function is the formation of a charge clamp *via* its H3 amino group that stabilizes the coactivator helix (12, 24). Lys284 H3 does not form direct polar contacts with other residues of the LBD. Therefore, it was expected that the conservative mutation of Phe450 to tyrosine and the mutation of Lys284 to glutamine, which at least partially preserves the aliphatic part of the Lys side chain, do not affect allosteric signaling across the dimer interface. The observation of almost unchanged RXRα homodimerization as well as heterodimerization with PPARγ supports this assumption.

Using the recruitment-incapable RXRα mutant, coactivator recruitment by PPARγ incorporated into the PPARγ:RXRα heterodimer can be studied independently and without interference. Mutant RXRα not competing for the coactivator allows concentrations of RXRα LBD high enough to ensure that throughout all conditions investigated the vast majority of PPARγ is incorporated into heterodimers with RXRα. The setup with sGFP as the FRET acceptor being coupled to RXRα further assures that only coactivator binding to the PPARγ:RXRα heterodimer is detected.

Our experiments also revealed limitations of the current setup. Coactivators and corepressors bind to different sites, and hence, the mutations introduced into the AF-2 of RXRα only block recruitment of coactivators but not corepressors. Indeed, a peptide corresponding to corepressor nuclear receptor corepressor 1 ID2 (aa 2251–2276) was still bound by apo RXRα incorporating the described AF-2 mutations (Fig. S17). Therefore, it is not possible to investigate corepressor binding utilizing the same assay setup. But the use of the developed RXRα AF-2 mutant will not be limited to investigations on cofactor recruitment by PPARγ. We have recently reported that the orphan nuclear receptor Nurr1 (NR4A2) responds to nonsteroidal anti-inflammatory drugs. Utilizing the dimer formation assay we were able to show that these drugs are capable of either promoting or weakening the formation of the Nurr1:Nurr1 homo- and/or the Nurr1:RXRα heterodimer, respectively (25). These findings demonstrate that the assay strategies outlined here will be of interest for studies on various NRs, especially those that partner with RXRα.

Finally, the set of assays presented in this study can serve as a toolkit for the development of novel types of NR modulators. Classical NR ligand development was focused on the extent of target gene expression or suppression (agonists, partial agonists, antagonists). Novel developments in the area of NR-related drug discovery comprises modulators of posttranslational modifications (26), selective degraders (ER degraders), or selective modulators (SEGRAs). The characterization and the understanding of the mode of action of these compounds will benefit from assays such as those presented in this study.

## Experimental procedures

### Cloning and mutagenesis of recombinant RXRα LBD and PPAR LBD fusion proteins

The coding sequence for human RXRα LBD and PPARα, PPARβ/δ, and PPARγ LBD was codon optimized for *E. coli* and purchased from GeneArt, respectively. For expression of fusion proteins with N-terminal green fluorescent protein (GFP), an expression construct based on pET29b was prepared. For this, the entire section between the original NdeI site and the fourth position following the His-Tag coding sequence of pET29b was replaced, hence, essentially leaving only the vector backbone unmodified. The section was replaced by a sequence encoding a restriction site for NcoI (overlapping with the start codon) and an open reading frame for Met-Gly-[His10-Tag]-Asp-Tyr-Asp-Ile-Pro-Thr-Thr-[TEV site]- super-folder GFP (sGFP) (27) followed by restriction sites for BamHI (in frame) and XhoI. The sequences coding for the LBDs of RXRα (UniProt entry: P19793-1, residues 226–462), PPARα (UniProt entry: Q07869-1, residues 198–468), PPARβ/δ (UniProt entry: Q03181-1, residues 170–441), or PPARγ isoform 2 (UniProt entry: P37231-1, residues 234–505), with the PPAR coding sequences each preceded by a single additional Gly as spacer, and each coding sequence followed by a stop codon were then introduced in frame between the afore inserted restriction sites for BamHI and XhoI.

For generation of biotinylated RXRα LBD or biotinylated PPARγ LBD the sGFP coding sequence was replaced by an Avi-tag (for position-specific labeling with biotin) that starts with the terminal Gly of the TEV site, respectively.

In order to generate PPARγ LBD devoid of any label after purification the sGFP coding sequence in pET29b-based plasmid was simply removed.

For generation of free RXRα LBD the pMal vector system (New England Biolabs, NEB) was used. In pMal-c2E, the section between the sequence encoding 10× Asparagine (Asn10) and the SalI restriction site was replaced with a sequence encoding Leu-Gly-Ile-Glu-Gly-Thr-[His8-Tag]-Pro-Gly-Thr-[TEV site] followed by restriction sites for BamHI (in frame) and XhoI. The latter were used for cloning RXRα (residues 223–462) followed by two stop codons. From this construct, a fusion protein is expressed with N-terminal maltose-binding protein (MBP) followed by an Asn10 linker, a His8-Tag, a cleavage site for TEV protease, and the RXRα LBD.

Mutations in RXRα LBD (V280T, K284E, V298T, F450Y, and/or E453R, and combinations thereof) were introduced by site-directed mutagenesis using pairs of forward and reverse oriented primers that held willfully modified positions in the middle of their overlapping sequences. Codon usage for *E. coli* was optimized for during mutagenesis.

### Expression

All proteins utilized in this study were coexpressed with GroEL/ES in order to support protein folding. For expression, *E. coli* T7 express cells (NEB) were cotransfected with pGro7 for coexpression of GroEL/ES (chaperone plasmid set; TaKaRa Bio, Inc) and one of the PPAR (pET with sGFP or Avi-tag) or RXRα (pET or pMal) expression constructs and selected on LB (Luria Broth) agar containing 34 μg/ml chloramphenicol and either 100 μg/ml ampicillin (for pMal) or 35 μg/ml kanamycin (for pET). Cultures in liquid LB medium containing the same antibiotics were grown at 37 °C and 180 rpm until absorbance at 600 nm (*A*_600_) reached 0.6 to 0.7. At this time point, expression of the chaperone GroEL/ES from pGro7 was induced with 1 g/l L(+)-Arabinose, temperature was reduced to 20 °C, and shaking to 120 rpm. About 20 to 30 min later *A*_600_ reached ~1, and expression of the target protein was induced by addition of 0.5 mM IPTG. The expression cultures were incubated overnight, harvested at 6000 rpm at 4 °C, and pellets were stored at −80 °C or processed right away. Pellets corresponding to 2 l of culture were resuspended in 50 ml buffer A (400 mM NaCl, 20 mM NaPi pH 7.8, 10% (w/v) glycerol and 20 mM ß-mercaptoethanol) supplemented with 20 mM imidazole. Cells were kept on ice and disrupted in the presence of 1 mM ATP, 750 Kunitz DNAse I and 250 Kunitz RNAse A (AppliChem), 2 to 5 mM MgSO_4_, and 1× EDTA-free complete protease inhibitor cocktail (F. Hoffmann-La Roche AG) by addition of lysozyme and ten passages through an Invensys APV-1000 homogenizer (APV Systems). Cell debris was removed by centrifugation at 16,500*g* for 20 min at 4 °C.

### Purification

For all proteins described the first step of purification was immobilized metal chromatography (IMAC) using columns packed with Ni Sepharose 6 Fast Flow resin on an ÄKTApurifier FPLC system (GE Healthcare). After washing for 15 column volumes with buffer A supplemented with 25 mM imidazole, the His-tagged fusion proteins were eluted with 300 mM imidazole.

All sGFP fusion proteins and PPARγ with only N-terminal His-tag-[TEV site] were then processed with His-tagged TEV protease (molar ratio 1:50) overnight while imidazole content was reduced to 10 mM by dialysis against buffer A in order to allow for reverse IMAC. The flow through was concentrated under 2 bar overpressure from nitrogen gas in an Amicon stirring cell equipped with a 10.000 MWCO membrane. We observed that sGFP-RXRα binds to the Ni resin even after the His-tag had been cleaved off as verified by a size shift on SDS-PAGE. Henceforth, for sGFP-RXRα the reserve IMAC step was omitted and the TEV digestion mixture was directly concentrated. Afterwards, concentrates were applied to size exclusion chromatography (SEC) using a 16/600 Superdex75 column equilibrated and run in high-glycerol HTRF buffer (25 mM Hepes pH 7.5, 150 mM KF, 10% (w/v) glycerol, 5 mM DTT). For PPARγ with/without sGFP-tag this resulted in a single monodisperse peak at an elution volume corresponding to monomeric protein. As expected, sGFP-RXRα forms dimers, which are in a concentration-dependent equilibrium with monomers. Owing to its size, this caused a substantial fraction of the protein to elute in the void volume of the SEC column. This, however, enabled us to remove the TEV protease and remnants of free sGFP as well as potentially defective protein that might not be capable of forming dimers. The fractions corresponding to dimeric sGFP-RXRα LBD were concentrated again and applied onto a 16/600 Superdex200. The concentration of the injected sample was lowered with respect to the previous SEC step in order to increase the fraction eluting as monomer, and only the corresponding fractions were later utilized. With this strategy, we ensured that all sGFP-RXRα proteins used in the various assays are capable of exchanging between monomer and dimer.

For purification of untagged RXRα from fusion proteins with MBP-His8-[TEV site] (pMal) lysis and the initial IMAC purification step were performed as before. Afterwards, the fusion proteins were processed overnight using a TEV protease that itself is fused to N-terminal MBP. This allowed removal of cleaved MBP, undigested target protein, and TEV protease by passage through a gravity column with Amylose High Flow resin (NEB). The flow through was concentrated as before and further purified by SEC on a 26/600 Superdex75 column run in high-glycerol HTRF buffer.

For generation of biotin-labeled proteins from the Avi-tag versions of RXRα or PPARγ the elution from IMAC was supplemented with His-tagged TEV protease (molar ratio approximately 1:50) and *E. coli* biotin ligase BirA (molar ratio approximately 1:10) for site-specific biotinylation of the Lys residue in the Avi-tag (28) in a dialysis setting against buffer A supplemented with 0.5 mM biotin, 0.5 mM ATP, and 5 mM MgCl_2_. After overnight incubation at 4 °C, the concentration of biotin was reduced to approximately ≤1 μM by repeated dialysis against buffer A. Thereafter, the solution was subjected to a column packed with 5 ml monomeric avidin UltraLink resin (Pierce Biotechnology Inc). Unlabeled proteins, TEV protease, and birA were removed by washing for ten column volumes with buffer A before biotin-labeled protein was eluted using buffer A supplemented with 2 mM biotin. The product was then concentrated and subjected to SEC using a 10/30 Superdex75 column equilibrated and run in high-glycerol HTRF buffer.

After the final purification step by SEC, proteins were aliquoted, flash frozen in liquid nitrogen, and stored at −80 °C. Proteins were not concentrated thereafter in order to prevent aggregation and assay artefacts.

### LBD:LBD dimer formation assay

The strength of LBD:LBD dimer formation and its modulation by ligands was investigated by HTRF with complex formation resulting in a gain in HTRF. The FRET donor complex formed from biotinylated NR LBD (final concentration 0.375 nM) and Terbium cryptate as streptavidin conjugate (Tb-SA; Cisbio; 0.75 nM) was kept constant while the concentration of sGFP-NR LBD as the FRET acceptor was varied starting with either 0.3 or 4 μM as the highest concentration and titrated with a dilution factor of 0.5. Free sGFP was added to keep the total sGFP content stable at 0.3 or 4 μM throughout the entire series in order to suppress artifacts from changes in degree of diffusion-enhanced FRET. Assay solutions were prepared in HTRF assay buffer supplemented with 0.1% (w/v) CHAPS as well as 1% DMSO with the test compounds at indicated concentrations or DMSO alone as the negative control. Samples were equilibrated at RT for 1 h before measurements.

### Coactivator recruitment on isolated NR LBD

Recruitment of coactivator-derived peptides to a NR LBD was also studied by HTRF. Peptides derived from the coactivators SRC1-2 (Steroid receptor coactivator 1) coactivator motif 2 [biotin-CPSSHSSLTERHKILHRLLQEGSPS] or CBP-1 (CREB-binding protein) coactivator motif 1 [biotin-NLVPDAASKHKQLSELLRGGSGS] featuring the coactivator consensus motif LxxLL and N-terminal biotin for stable coupling to streptavidin were purchased (Eurogentec GmbH). The concentration of the test compounds was varied starting at 100 μM as the highest concentration and from there titrated with a dilution factor of 0.4. The respective fusion protein of FRET acceptor sGFP and the NR LBD was applied at 100 nM, and the FRET donor complex formed from either SRC1 or CBP derived peptide and Tb-SA at 12 nM. Assay solutions were prepared in HTRF assay buffer supplemented with 0.1% (w/v) CHAPS as well as 1% DMSO. When testing antagonists on PPARγ the solution of sGFP- PPARγ LBD and the FRET donor complex was supplemented with 1 μM rosiglitazone, which corresponds to the EC_80_ concentration of rosiglitazone in activation of PPARγ. In both settings the samples were equilibrated at RT for 2 h before measurements.

### Coactivator recruitment by PPARγ LBD when accompanied by its dimer partner RXRα

The CBP recruitment by PPARγ LBD in the context of the PPARγ LBD forming a heterodimer with the RXRα LBD was again studied in a HTRF assay system. One-hundred nanomolar PPARγ LBD without sGFP-tag was supplemented with 2 μM sGFP fusion protein with mutant RXRα LBD incapable of recruiting coactivators itself. The sGFP-tag again served as the FRET acceptor to 12 nM FRET donor complex composed of CBP-derived peptide coupled to Tb-SA. We varied the concentration of the test compounds starting at 100 μM and titrated them with a dilution factor of 0.4. All solutions were prepared in HTRF assay buffer supplemented with 0.1% (w/v) CHAPS as well as 1% DMSO. In order to test the antagonistic effect of compounds on PPARγ in the context of the dimer with RXRα the solution of PPARγ LBD, sGFP-RXRα LBD, and the FRET donor complex was supplemented with 1 μM rosiglitazone. This concentration corresponds to the EC_80_ concentration of rosiglitazone activation. Before measurements the samples were equilibrated at RT for 2 h.

### Plates and assay format

All HTRF experiments were conducted with a final assay volume of 20 μl in white nontreated polystyrene shallow-well 384-well plates of the type Nunc 264706 (ThermoFisher). For protection from evaporation and exposure to light during incubation plates were sealed with adhesive aluminum foil (4TI-0550; 4titude Ltd).

### Measurement of HTRF

After incubation at RT the fluorescence intensities (FIs) at 520 nm (acceptor) and 620 nm (donor reference) after excitation at 340 nm were recorded either on a Tecan Infinite F200 or a Tecan SPARK (Tecan Deutschland GmbH), the latter with enhanced fluorescence module, and filter-based measurement routine on both devices. Measurements were performed with 50 flashes, an integration time of 400 μs, and a lag time of 100 μs. The gain was always set to optimal, since experiments that had to be compared were always measured on the same plate.

### Calculation of HTRF

In order to obtain the dimensionless HTRF signal FI520 nm was divided by FI620 nm and multiplied with 10,000 as shown below.

$$HTRF=\frac{acceptor\;fluorescence\cdot 10000}{donor\;fluorescence}$$

### Curve fitting

Data analysis was conducted using GraphPad Prism, Version 8 (GraphPad Software, Inc) utilizing the dose-response stimulation or inhibition protocol with four parameter curve fit for determination of bottom and top plateaus, EC_50_ or IC_50_, as well as the hill slope. EC_50_ or IC_50_ values as well as hill slopes were compared using a sum-of-squares F-test.

### Statistics

The cell-free cofactor recruitment and dimer formation experiments were performed in general with three technical replicates (N = 3), or four (N = 4) if indicated. Key experiments that ask how certain ligands influence homo- or heterodimer formation have been conducted in three independent experiments (n = 3).

The 2D titration presented in Figure 14 was conducted in two technical replicates per data point (N = 2).

### Calculation of apparent *K*_*d*_

In order to calculate the *K*_*d*_ of dimer formation we used the following equation, with [g.p.] being the concentration of the GFP-labeled protein at the inflection point of the binding curve.

$$Kd=\frac{\frac{1}{2}\;\left[b.\;p.\right]\cdot \left(\left[g.p.\right]-\frac{1}{2}\left[b.p.\right]\right)}{\frac{1}{2}\left[b.p.\right]}$$

[b.p.] is the concentration of the biotin-labeled protein that is constant throughout the experiment. It is assumed that, at the inflection point, 50% of b.p. forms a dimer with GFP-labeled protein and that the remaining 50% of b.p. is monomeric and available for complex formation. The molecular structure of the streptavidin tetramer puts the suggestion close that the biotin-binding sites are pairwise located in such close proximity that of each pair only one at a time can be occupied by a biotin-labeled Avi-tag. The concentrations of the b.p. were in all experiments fairly below the *K*_*d*_ of dimer formation, respectively. Henceforth, we assume that, once bound to Tb-SA, the b.p. is present as monomeric LBD bound at opposing poles on the streptavidin tetramer. Accordingly, in the dimerization assays Tb-SA was used in 2-fold molar excess of streptavidin monomers relative to b.p.

### Cloning of plasmids for hybrid reporter gene assays

The plasmids pFR-Luc (Stratagene; transactivation reporter) and pRL-SV40 (Promega; internal control) were used for hybrid reporter gene assays in combination with the following plasmids. The plasmid pFA-CMV-hRXRα-LBD wildtype has previously been described (29). From this plasmid a fusion protein is expressed that consists of the Gal4 DBD, and the hinge region and LBD of human RXRα (aa 225–462). The AF-2 mutations (V280T, K284E, V298T, F450Y, and E453R) were introduced in order to generate the pFA-CMV-hRXRα-LBD mutant.

To enable formation of the PPARγ:RXRα heterodimer to be detected as a gain in transactivation of firefly expression we cloned the plasmid pFTI-CMV-PPARγ-LBD. In the original plasmid pFA-CMV (fusion trans-activator plasmid; agilent #219036; PathDetect system) the section encoding the Gal4-DBD (that starts with the eighth codon of the CMV controlled open reading frame) was replaced by a DNA sequence coding for VP16 (α-trans inducing factor; UniProt P06492; aa 413–490) followed by a Gly-Ser linker. The resulting plasmid was named pFTI-CMV (fusion trans-inducing factor plasmid). Into this plasmid the native cDNA sequence for human PPARγ (aa 204–505) was inserted between the restriction sites for BamHI and XbaI within the original multiple cloning site. Expression of the fusion protein MDYKDDVAST-[VP16 (aa 413–490)]-SSGGGGSSGGS-[PPARγ LBD (aa 204–505)] is under the control of the CMV promoter.

In order to detect (primarily) recruitment of steroid receptor coactivator 1 (SRC1) to ligand-activated RXR we cloned SRC1 motif 2 (SRC1-2) into pFTI-CMV. The N-terminal cysteine of SRC1-2 was replaced with serine. The plasmid pFTI-CMV-SRC1-2 conveys expression of the fusion protein MDYKDDVAST-[VP16 (aa 413–490)]-SSGGGGSSGGS-[SRC1-2; S PSSHSSLTERHKILHRLLQEGSPSD].

### Reporter gene assays

pFR-Luc (Stratagene) was used as reporter plasmid and pRL-SV40 (Promega) for normalization of transfection efficiency.

#### Assay procedure

HEK293T cells (German Collection of Microorganisms and Cell Cultures [DSMZ]) were cultured in Dulbecco’s modified Eagle’s medium, high glucose, supplemented with 10% fetal calf serum, sodium pyruvate (1 mM), penicillin (100 U/ml), and streptomycin (100 μg/ml) at 37 °C and 5% CO_2_. Cells were seeded in 96-well plates (3 × 10^4^ cells/well) 24 h prior to transfection. Before transfection, the medium was changed to Opti-MEM without supplements. Transient transfection with the indicated plasmid mixtures in combination with pFR-Luc and pRL-SV40 was performed using Lipofectamine LTX reagent (Invitrogen) according to the manufacturer’s protocol.

Five hours after transfection, the medium was changed to Opti-MEM supplemented with penicillin (100 U/ml) and streptomycin (100 μg/ml). In experiment series involving stimulation with SR11237 the medium now additionally contained 0.1% DMSO and the respective concentration of SR11237 or 0.1% DMSO alone as control. Each experiment was performed independently at least five times. Following overnight (14–16 h) incubation, cells were assayed for luciferase activity using the Dual-Glo Luciferase Assay System (Promega) according to the manufacturer’s protocol. Luminescence was measured with a Spark 10 M luminometer (Tecan Group Ltd). Normalization of transfection efficiency and cell growth was done by division of firefly luciferase data by Renilla luciferase data and multiplying the value by 1000 resulting in relative light units.

These cellular experiments were performed with five independent biological repeats (n = 5).

## Data availability

The data that support the findings of this study are contained within the article and the supporting information. All data are available from the corresponding author upon reasonable request.

## Supporting information

This article contains supporting information.

## Conflict of interest

The authors declare that they have no conflicts of interest with the contents of this article.
