# Supplementary material for: Heterodimer formation with retinoic acid receptor RXRα modulates coactivator recruitment by peroxisome proliferator-activated receptor PPARγ
Source: J Biol Chem. 2021 May 31;297(1):100814. doi: 10.1016/j.jbc.2021.100814 (PMC8258697; doi:10.1016/j.jbc.2021.100814)
Supplement: Supplemental Figures S1–S17 [file mmc1.pdf]

Supporting Information

for

Heterodimer formation with retinoic acid receptor RXRα modulates coactivator recruitment by peroxisome proliferator-activated receptor PPARγ

Whitney Kilu[1], Daniel Merk[1], Dieter Steinhilber[1,2], Ewgenij Proschak[1,2], Jan Heering[2]*

[1] Institute of Pharmaceutical Chemistry, Goethe University Frankfurt, Max-von-Laue-Str. 9, D-60438 Frankfurt, Germany

[2] Fraunhofer Institute for Translational Medicine and Pharmacology ITMP, Theodor-Stern-Kai 7, D-60596 Frankfurt, Germany

*To whom correspondence should be addresses: Jan Heering, Fraunhofer Institute for Translational Medicine and Pharmacology ITMP; Jan.Heering@ime.fraunhofer.de; Tel. +49 69 79829204

Running title: PPARγ activation in the context of the heterodimer with RXRα

Keywords: PPARγ, RXRα, heterodimer, homodimer, homogeneous time-resolved FRET (HTRF), cofactor recruitment, allostery, drug target

SI method 1:	Pulldown assay with PPAR proteins captured by biotin-labeled RXRα LBD	S-3
SI method 2:	Isothermal titration calorimetry	S-3
Figure S1:	Validation of intact dimer formation capability of PPARγ protein preparations via pulldown assay.	S-4
Figure S2:	Validation of intact dimer formation capability of PPARα and PPARδ protein preparations via pulldown assays.	S-4
Figure S3:	Control experiments for heterodimer formation assays with titrated sGFP-PPAR LBD.	S-5
Figure S4.	Control experiments for heterodimer formation assays with titrated sGFP-RXRα LBD.	S-6
Figure S5.	Ligand dependent recruitment of coactivator CBP-1 by biotin labeled PPARγ LBD coupled to Tb-SA.	S-7
Figure S6:	Effect of PPARγ antagonists on PPARγ:RXRα heterodimer formation.	S-8
Figure S7:	Evaluation of protein concentration as a factor for investigation of rosiglitazone dependent CBP-1 coactivator recruitment to the PPARγ LBD	S-9
Figure S8:	Analysis of LBD:LBD heterodimer formation between PPARγ and all RXRα mutants generated in comparison to wildtype.	S-10

Figure S9:	Schematic depiction of the cell-based Gal4-RXRα VP16-PPARγ heterodimer formation assay	S-11
Figure S10:	Assessment of transactivation activity of VP16-SRC1 alone and in combination with Gal4-RXRα with no agonist being present.	S-12
Figure S11:	ITC experiment for verification of SR11237 binding to the RXRα wildtype LBD	S-13
Figure S12:	ITC experiment for verification of SR11237 binding to the RXRα total mutant LBD	S-14
Figure S13:	Recruitment of coactivator CBP-1 by PPARγ LBD in the context of the heterodimer with the RXRα LBD.	S-15
Figure S14:	Comparison of recruitment of coactivator CBP-1 by PPARγ LBD either being present as monomeric LBD or in the context of the LBD:LBD heterodimer with RXRα LBD.	S-16
Figure S15:	Effect of RXRα agonists SR11237 on RXRα homodimer formation and comparison between RXRα wildtype and recruitment incapable mutant.	S-17
Figure S16:	Modulation of RXRα LBD homodimer formation by Tetrac.	S-18
Figure S17:	Agonist dependent displacement of NCOR-1 corepressor from apo RXRα LBD mutant.	S-19

SI method 1: LBD pulldown

In order to verify proper dimer formation capability of the majority of the protein preparation a pulldown experiment was performed. Therefore, small centrifugal filter columns (nylon membrane with 0.2 µM pore size; VWR) were loaded with 20 µl of Pierce® high capacity streptavidin agarose beads (Thermo Scientific) equilibrated in HTRF buffer [25 mM HEPES pH 7.5, 150 mM KF, 10% (w/v) glycerol, 5 mM DTT (fresh)], then 20 µl of a mixture of biotin coupled RXRα LBD (4 µg) and the respective PPAR LBD (1.4 µg) was applied. After incubation for 10 min at room temperature followed by centrifugation for 2 min and 900 x g the input mixture as well as the flowthrough was analyzed via SDS PAGE. Gels were scanned using an Li-Cor Odyssey imager (LI-COR® Biosciences GmbH, Germany). Missing bands in comparison to the input control were interpreted in two different ways. For RXRa it showed that the streptavidin beads were now coated with the biotin-labeled RXRa LBD. In the case of the PPAR proteins a missing band was evidence of intact LBD:LBD dimer formation between RXRa and the particular PPAR LBD.

SI method 2: Isothermal titration calorimetry

The capability of the RXRα-LBDs to bind the agonist SR11237 was verified via isothermal titration calorimetry (ITC) conducted on an TA Instruments Affinity ITC (TA Instruments, New Castle, Delaware, USA). Recombinant (tag-free) RXRα LBD wt or mutant (120 µM) and SR11237 (10 µM) were dissolved in HTRF buffer [25 mM HEPES pH 7.5, 150 mM KF, 10% (w/v) glycerol, 5 mM DTT (fresh)] supplemented with 1% DMSO (v/v). The ITC instrument was adjusted to 25°C and the stirring rate was set to 75 rpm. Compound solution was filled into the reaction cell (178 µl cell volume) and a solution with one of the RXRα LBDs was titrated (inverse titration). The first injection had a reduced volume of 0.5 µL and was followed by 25 injections of 2.0 µL. An interval of 300 s was maintained between individual injections. The heats of dilution resulting from titrating one of the RXRα LBD proteins into the cell containing only buffer were recorded separately and subtracted from the raw ITC data obtained with compound. Data were analyzed using the NanoAnalyze software package (version 3.7.5). In order to fit reaction enthalpy (ΔH), binding affinity constant (K_d), and stoichiometry (n) an independent binding model was used. Free energy change (ΔG) was calculated using the equation

$$\Delta G = -RT \cdot \ln K$$

and from there the entropy (ΔS) was calculated from

$$\Delta G = \Delta H - T\Delta S$$

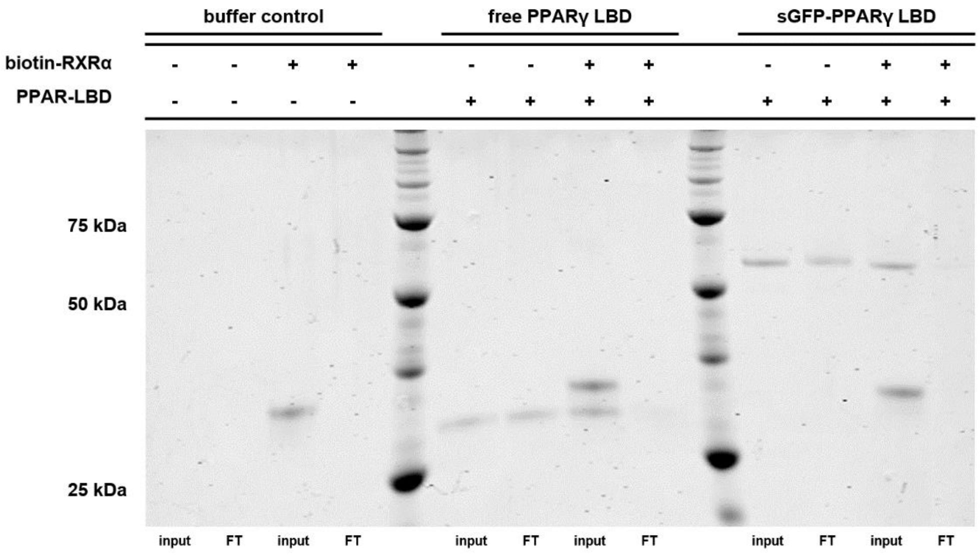

Figure S1. Validation of intact dimer formation capability of PPARγ protein preparations via pulldown assays. Preparations of both free PPARγ LBD and sGFP-PPARγ LBD were tested in a pulldown experiment using RXRα coated streptavidin-agarose beads. Comparison of the input and the flowthrough (FT) demonstrates the retention of the respective PPARγ LBD through formation of the heterodimer complex with RXRα. Controls prove both proper coating of the streptavidin-agarose beads with the biotin coupled RXRα LBD, and no unspecific binding of the PPARγ LBDs to the column material.

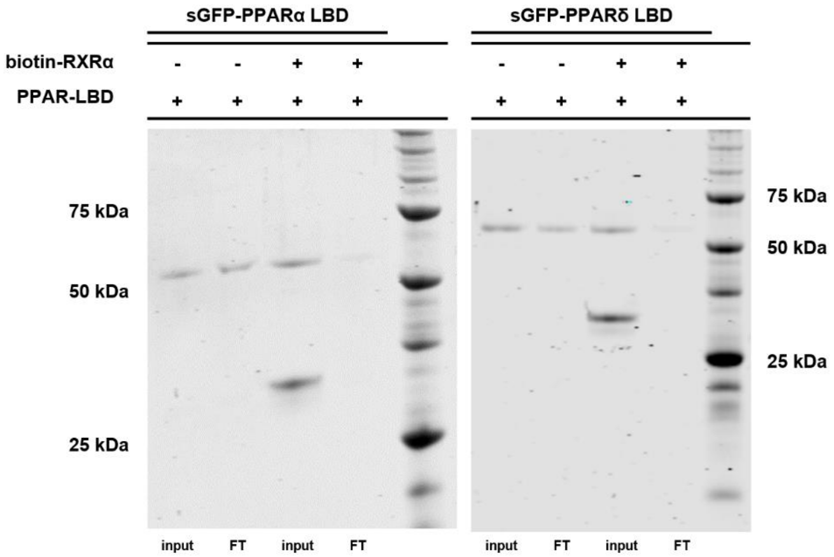

Figure S2. Validation of intact dimer formation capability of PPARα and PPARδ protein preparations via pulldown assays. Preparations of both sGFP-PPARα LBD, and sGFP-PPARδ LBD were tested in a pulldown experiment using RXRα coated streptavidin-agarose beads. Comparison of the input and the flowthrough (FT) demonstrates the retention of the respective PPAR LBD through formation of the heterodimer complex with RXRα. Controls prove both proper coating of the streptavidin-agarose beads with the biotin coupled RXRα LBD and no unspecific binding of the PPAR LBDs to the column material.

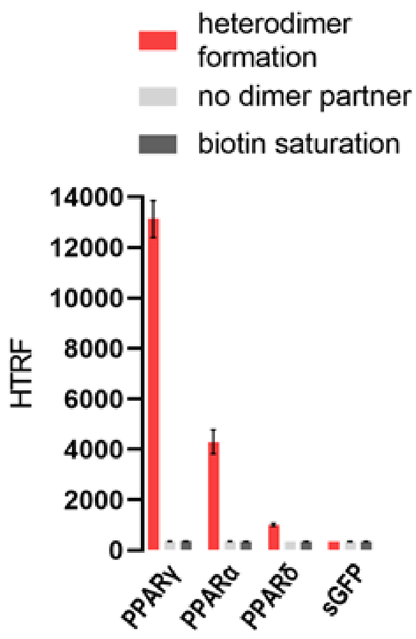

	hetero dimer formation	no dimer partner	biotin saturation
**sGFP-PPAR LBD**	+	+	+
**biotin labeled RXRα LBD**	+	-	+
**Tb-SA**	+	+	+
**1.2 µM biotin**	-	-	+

Figure S3. Control experiments for heterodimer formation assays with titrated sGFP-PPAR LBD. LBD of RXRα was labeled with biotin via an N-terminal Avi-tag and coupled to Tb-SA (12 nM RXRα and 12 nM Streptavidin monomers, respectively). 12 nM of the respective sGFP-PPAR LBD was present in all experiments. Binding of sGFP-PPAR to RXRα results in close proximity to Tb-SA and an increase in HTRF in comparison to controls. In samples with biotin saturation the coupling of RXRα to Tb-SA was blocked with an 100-fold molar excess of biotin. This uncoupled heterodimer formation from the recruitment of sGFP into a complex with Tb-SA. HTRF signals of these controls were comparable to controls with no RXRα being present (no dimer partner). Data are the mean ± SD; N = 4.

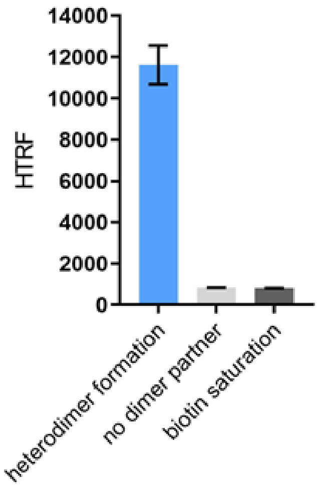

	hetero dimer formation	no dimer partner	biotin saturation
**sGFP-RXRα LBD**	+	+	+
**biotin labeled PPARγ LBD**	+	-	+
**Tb-SA**	+	+	+
**1.2 µM biotin**	-	-	+

Figure S4. Control experiments for heterodimer formation assays with titrated sGFP-RXRα LBD. LBD of PPARγ was labeled with biotin via an N-terminal Avi-tag and coupled to Tb-SA (12 nM PPARγ and 12 nM Streptavidin monomers, respectively). 12 nM of sGFP-RXRα LBD was present in all experiments. Binding of sGFP-RXRα to PPARγ results in close proximity to Tb-SA and an increase in HTRF in comparison to controls. When the coupling of PPARγ to Tb-SA was blocked with an 100-fold molar excess of biotin this uncoupled heterodimer formation from the recruitment of sGFP into a complex with Tb-SA. The HTRF signal of this control was comparable to a second control with no PPARγ being present (no dimer partner). Data are the mean ± SD; N = 3.

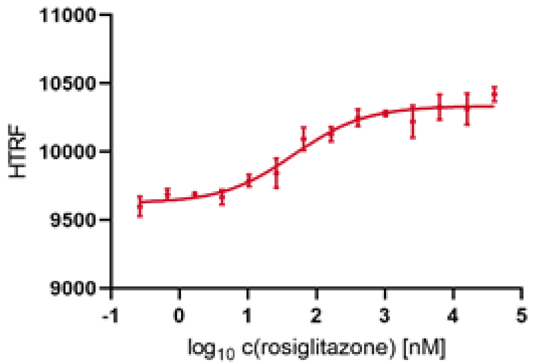

Figure S5. Ligand dependent recruitment of coactivator CBP-1 by biotin labeled PPARγ LBD coupled to Tb-SA. PPARγLBD was labeled with biotin via an N-terminal Avi-tag and subsequently coupled to streptavidin labeled with Terbium cryptate (Tb-SA). The latter was utilized as the FRET donor fluorophore. Reference agonist rosiglitazone was titrated against 100 nM fluorescein (FITC) coupled CBP-1 cofactor peptide (CREB-binding protein; coactivator motif 1; FITC-AASKHKQLSELLRGGSGSS; ThermoFisher; PV4596), 12 nM biotin coupled PPARγ LBD and 12 nM Tb-SA. HTRF measurements were performed after 1 h incubation at room temperature. Reader settings were the same as described for experiments with sGFP instead of FITC serving as the FRET acceptor. Data are the mean ± SD; N = 3. R^2 equals 93 %. The EC_{50} for rosiglitazone was determined at 48 ± 11 nM.

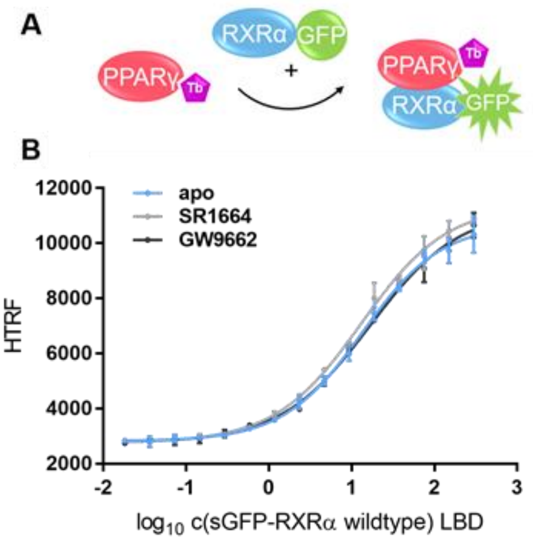

	apparent K_d
apo	13 ± 0.9 nM
SR1664	13 ± 0.9 nM
GW9662	16 ± 1.3 nM

Figure S6. Effect of PPARγ antagonists on PPARγ:RXRα heterodimer formation. A, schematic representation of the heterodimer formation assay with PPARγ LBD being coupled to the FRET donor. B, sGFP-RXRα LBD was titrated against 0.375 nM biotinylated PPARγ LBD and 0.75 nM Tb-SA with either 1 µM PPARγ irreversible antagonist GW9662 (black), non-agonist SR1664 (grey) or no ligand at all (light blue). HTRF measurements were performed after 1 h incubation at room temperature. Adding up free sGFP the total concentration of sGFP was kept constant at 0.3 µM throughout the entire experiment. Data are the mean ± SD; N = 3. R^2 for each curve equals >99 %.

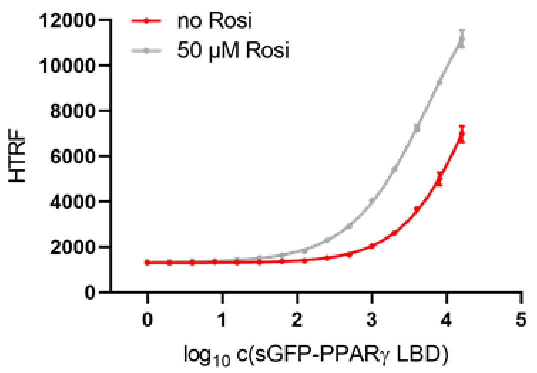

Figure S7. Evaluation of protein concentration as a factor for investigation of rosiglitazone dependent CBP-1 coactivator recruitment to the PPARγ LBD. Refer to Figure 4 A in main manuscript for schematic presentation of the PPARγ monomer cofactor recruitment assay. PPARγ LBD as a fusion protein with N-terminal sGFP was titrated against 12 nM Tb-SA, 12 nM biotinylated CBP-1 cofactor peptide. Recruitment of CBP-1 to the PPARγ LBD resulted in an increase in HTRF. PPARγ was activated with 50 µM of its reference agonist rosiglitazone and compared to apo (DMSO control). HTRF measurements were performed after 1 h incubation at room temperature. Data are the mean ± SD; N = 4.

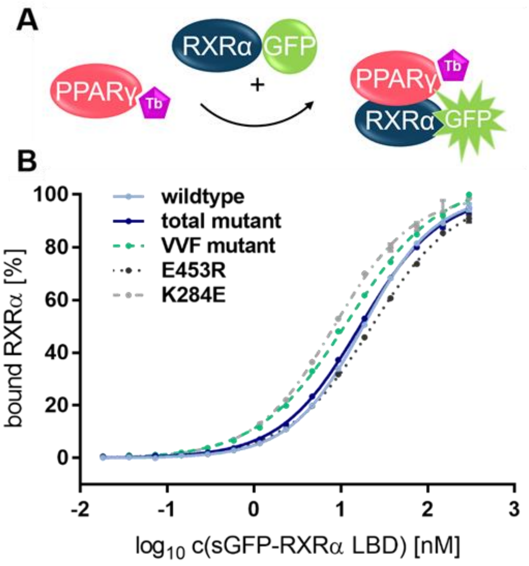

	apparent K_d
wildtype	18 ± 0.2 nM
total mutant	16 ± 0.4 nM
VVF mutant	12 ± 0.7 nM
E453R	23 ± 0.7 nM
K284E	8 ± 0.3 nM

Figure S8. Analysis of LBD:LBD heterodimer formation between PPARγ and all RXRα mutants generated in comparison to wildtype. A, schematic representation of the heterodimer formation assay with PPARγ LBD being coupled to the FRET donor. B. sGFP-RXRα LBD wildtype (light blue), mutant E453R (black dotted), mutant K284E (grey dotted), VVF mutant (V280T, V298T, and F450Y) (green dotted), or total mutant (dark blue) harboring all five mutations (V280T, K284E, V298T, F450Y, and E453R) was titrated against 0.375 nM biotinylated PPARγ LBD and 0.75 nM Tb-SA. Adding up free sGFP the total concentration of sGFP was kept constant at 0.3 µM throughout the entire experiment. Data are the mean ± SD; N = 3. R^2 for each curve equals >99 %.

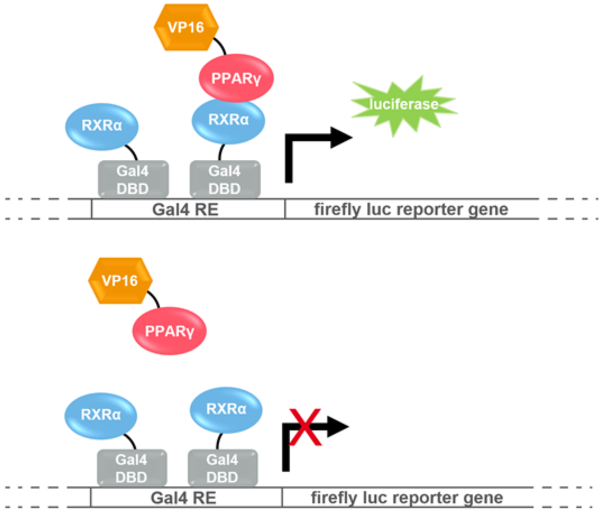

Figure S9. Schematic depiction of the cell-based Gal4-RXRα VP16-PPARγ heterodimer formation assay. RXRα is expressed as a fusion protein with Gal4 DBD. The latter guides RXRα to the Gal4 DNA response elements in the promoter region upstream of the firefly gene in plasmid pFR-Luc. PPARγ is expressed as a fusion protein with VP16, a strong trans-inducer of transcription. Formation of the RXRα:PPARγ heterodimer results in recruitment of VP16 to the Gal4 response elements, and subsequently activates expression of firefly luciferase (Luc). Renilla Luc is constitutively expressed from pRL-SV40 and is utilized as an internal control for transfection and toxicity. Luminescence of firefly Luc devided by the luminescence of renilla Luc is a measure for strength of transactivation resulting from co-expression of the respective NR fusion proteins. When compared to appropriate controls, this strategy can be used to detect dimer formation between VP16-PPARγ and Gal4-RXRα.

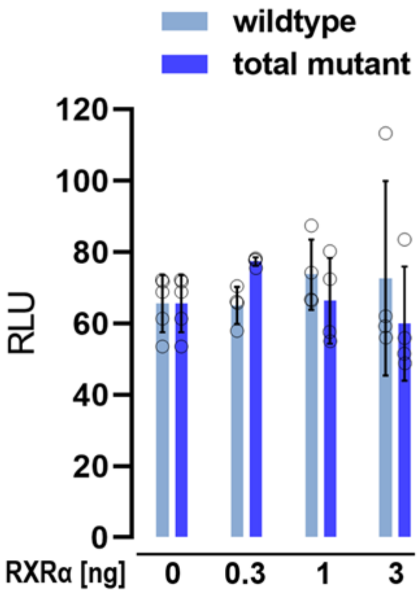

Figure S10. Assessment of transactivation activity of VP16-SRC1 alone and in combination with Gal4-RXRα with no agonist being present. HEK293T cells were co-transfected with 1 ng of the VP16-SRC1-2 plasmid, and various amounts of either Gal4-RXRα wildtype or Gal4-RXRα total mutant plasmid, always in combination with the plasmids for firefly reporter and renilla luciferase. Luciferase fluorescence was detected using the Dual-Glo™ Luciferase Assay System (Promega). Data are the mean ± SD; n = 5.

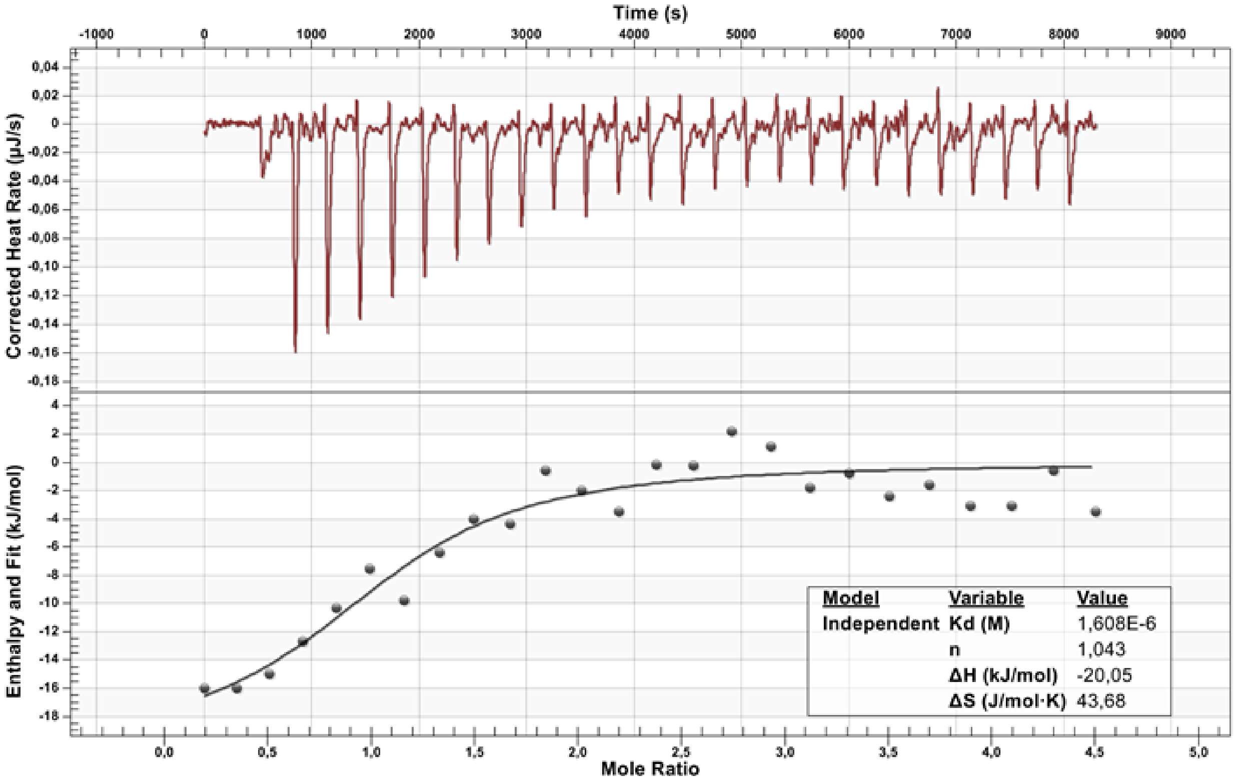

Figure S11. ITC experiment for verification of SR11237 binding to the RXRα wildtype LBD. Recombinant RXRα wildtype LBD (120 µM) was placed into the syringe and titrated to SR11237 (10 µM). 25 injections of 2 µl were performed after an initial injection of 0.5 µl. An interval of 300 s was maintained between individual injections and the experiments were performed at 25°C and a stirring rate of 75 rpm. The heats of dilution resulting from titrating of protein into buffer were recorded separately and subtracted. Thereafter, data were fitted to an independent binding model.

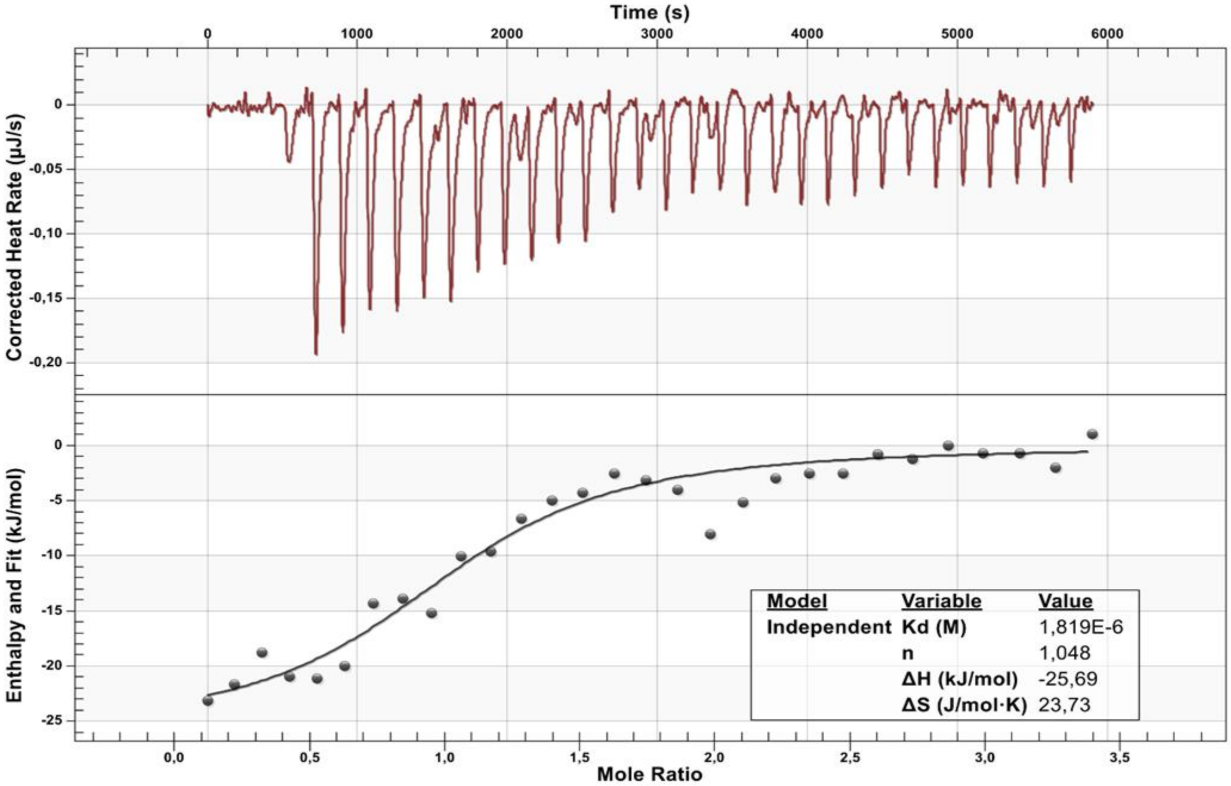

Figure S12. ITC experiment for verification of SR11237 binding to the RXRα total mutant LBD. Recombinant RXRα LBD (120 µM) harboring the five mutations that prevent recruitment (V280T, K284E, V298T, F450Y, and E453R) was titrated to 10 µM SR11237. 25 injections of 2 µl were performed after an initial injection of 0.5 µl. An interval of 300 s was maintained between individual injections and the experiments were performed at 25°C and a stirring rate of 75 rpm. The heats of dilution resulting from titrating of protein into buffer were recorded separately and subtracted. Thereafter, data were fitted to an independent binding model.

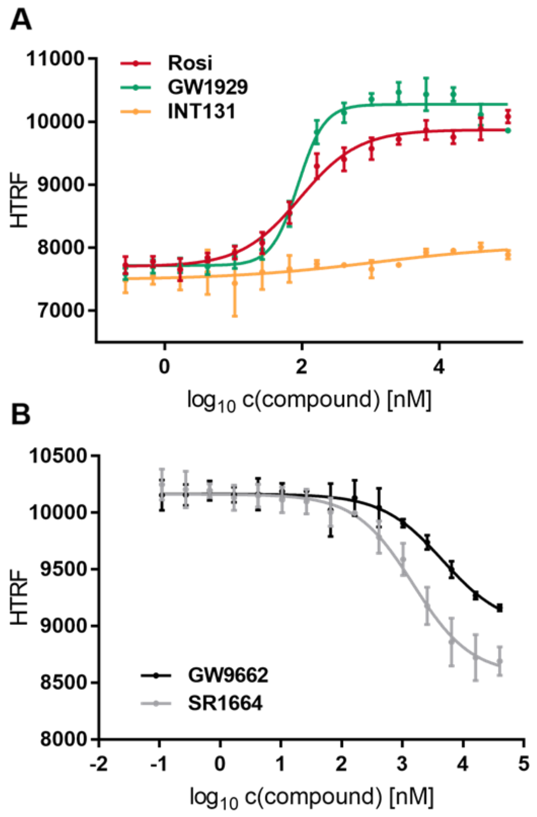

Figure S13. Recruitment of coactivator CBP-1 by PPARγ LBD in the context of the heterodimer with the RXRα LBD. 100 nM PPARγ LBD was incubated with 2 µM sGFP-RXRα LBD harboring mutations (V280T, K284E, V298T, F450Y, and E453R) that block coactivator recruitment by RXRα. The excess of RXRα ensures that throughout the experiments the main share of PPARγ is being incorporated into the PPARγ-RXRα heterodimer. Recruitment of biotin-labeled CBP-1 coactivator peptide (12 nM) coupled to Tb-SA (12 nM) is detected as an increase in HTRF. A, CBP-1 recruitment by PPARγ is modulated by titration of PPARγ full agonist GW1929 (green), partial agonist INT131 (yellow), or reference agonist rosiglitazone (red). B, CBP-1 recruitment is stimulated with constant 1 µM rosiglitazone and challenged by titration of GW9662 (black), or SR1664 (grey). Data are the mean ± SD; N = 3. R^2 for each curve equals >97 %.

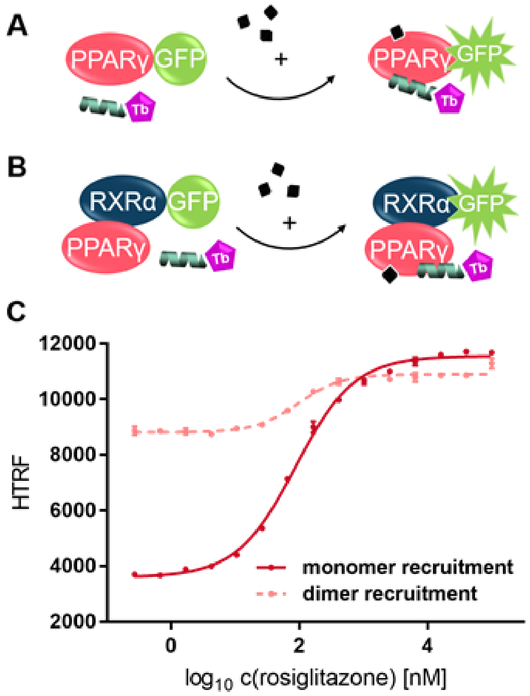

Figure S14. Comparison of recruitment of coactivator CBP-1 by PPARγ LBD either being present as monomeric LBD or in the context of the LBD:LBD heterodimer with RXRα LBD. A, schematic presentation of monomer recruitment assay. B, schematic presentation recruitment of coactivator by PPARγ in the context of the heterodimer with mutant RXRα. C, for investigation of coactivator recruitment by isolated monomeric PPARγ LBD 100 nM sGFP-PPAR was presented. Recruitment by PPARγ in the context of the heterodimer with RXRα was investigated on 100 nM free PPARγ LBD of which the main share is incorporated into the LBD:LBD heterodimer with sGFP-RXRα LBD (2 µM; 20-fold molar excess). The RXRα LBD harbors mutations (V280T, K284E, V298T, F450Y, and E453R) that block coactivator recruitment by RXRα. In either case recruitment of biotin-labeled CBP-1 coactivator peptide (12 nM) coupled to Tb-SA (12 nM) was detected as an increase in HTRF. The recruitment was stimulated by titration of rosiglitazone. Data are the mean ± SD; N = 4. R^2 equals 97.4% for dimer recruitment, and 99.7% for monomer recruitment.

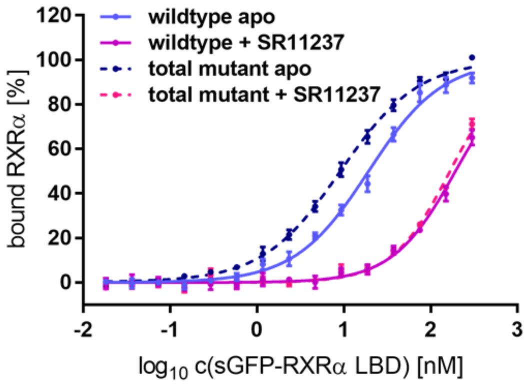

	apparent K_d	
	apo	+SR11237
wildtype	19 ± 1 nM	190 ± 8 nM
total mutant	9 ± 0.4 nM	167 ± 8 nM

Figure S15. Effect of RXRα agonists SR11237 on RXRα homodimer formation and comparison between RXRα wildtype and recruitment incapable mutant. sGFP-RXRα LBD was titrated onto 0.375 nM biotinylated RXRα LBD coupled to Tb-SA (0.75 nM Streptavidin subunits). For both wildtype RXRα LBD as well as RXRα mutant (V280T, K284E, V298T, F450Y, and E453R) the experiment was conducted on apo protein, or with constant 10 µM SR11237 being present. Adding up free sGFP the total concentration of sGFP was kept constant at 0.3 µM throughout the entire experiment.

Due to the substantial increase in the K_d resulting from treatment with SR11237 the respective curves did no longer reach the upper plateau corresponding to saturation binding. Therefore, the upper plateau (HTRF signal) from the respective experiment with only DMSO (apo) was set as 100% bound for curve fitting and calculation of apparent K_d of dimer formation. Data are the mean ± SD; N = 3. R^2 for each curve equals >98%.

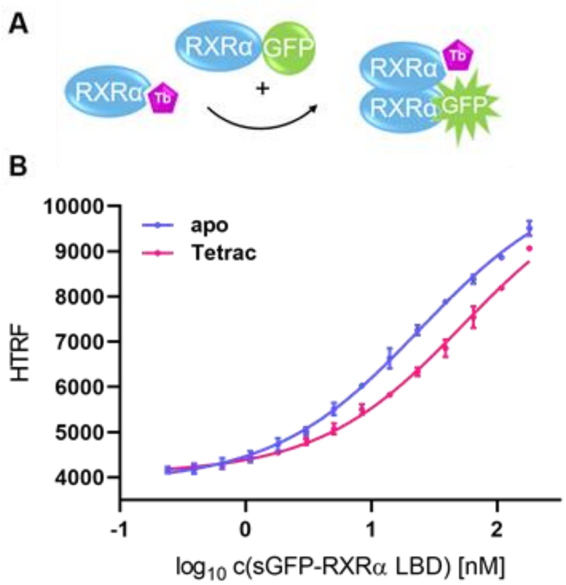

	apparent K_d
apo	28 ± 5 nM
Tetrac	50 ± 2 nM

Figure S16. Modulation of RXRα LBD homodimer formation by Tetrac. A, schematic representation of the homodimer formation assay with RXRα LBD being coupled to the FRET donor and titrated with sGFP-RXRα. B, sGFP-RXRα LBD was titrated against 0.375 nM biotinylated RXRα LBD and 0.75 nM Tb-SA. Adding up free sGFP the total concentration of sGFP was kept constant at 0.3 µM throughout the entire experiment. The effect of a constant concentration of 10 µM Tetrac was investigated in comparison to no ligand. Data are the mean ± SD; N = 3. R^2 for each curve equals >99%. The graph shows one representative experiment out of three independent experiments (n = 3) each conducted with three technical replicates (N = 3). The table reports the mean of apparent K_d ± SD calculated based on the values derived from the entire set of three independent experiments, respectively.

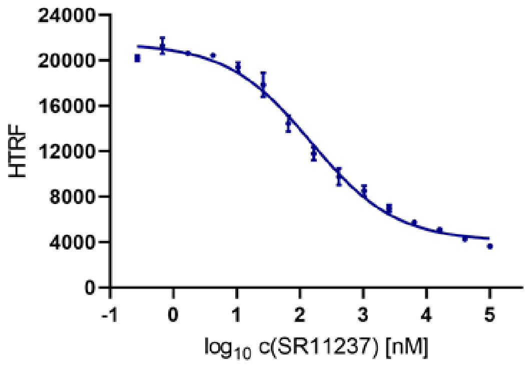

Figure S17. Agonist dependent displacement of NCOR1 corepressor from apo RXRα LBD mutant. SR11237 was titrated against 12 nM Tb-SA, 12 nM biotinylated NCOR1 ID2 (aa 2251-2276) and 100 nM sGFP-RXRα mutant LBD harboring mutations (V280T, K284E, V298T, F450Y, and E453R) that block coactivator recruitment. A reduction in HTRF corresponds to a reduction in complex formed between NCOR1 and RXRα. Data are the mean ± SD; N = 3. R^2 for each curve equals >98%..
